# Supplementary figures and images for: Phase separation of the PRPP amidotransferase into dynamic condensates promotes de novo purine synthesis in yeast
Source: PLoS Biol. 2025 Apr 10;23(4):e3003111. doi: 10.1371/journal.pbio.3003111 (PMC12017579; doi:10.1371/journal.pbio.3003111)

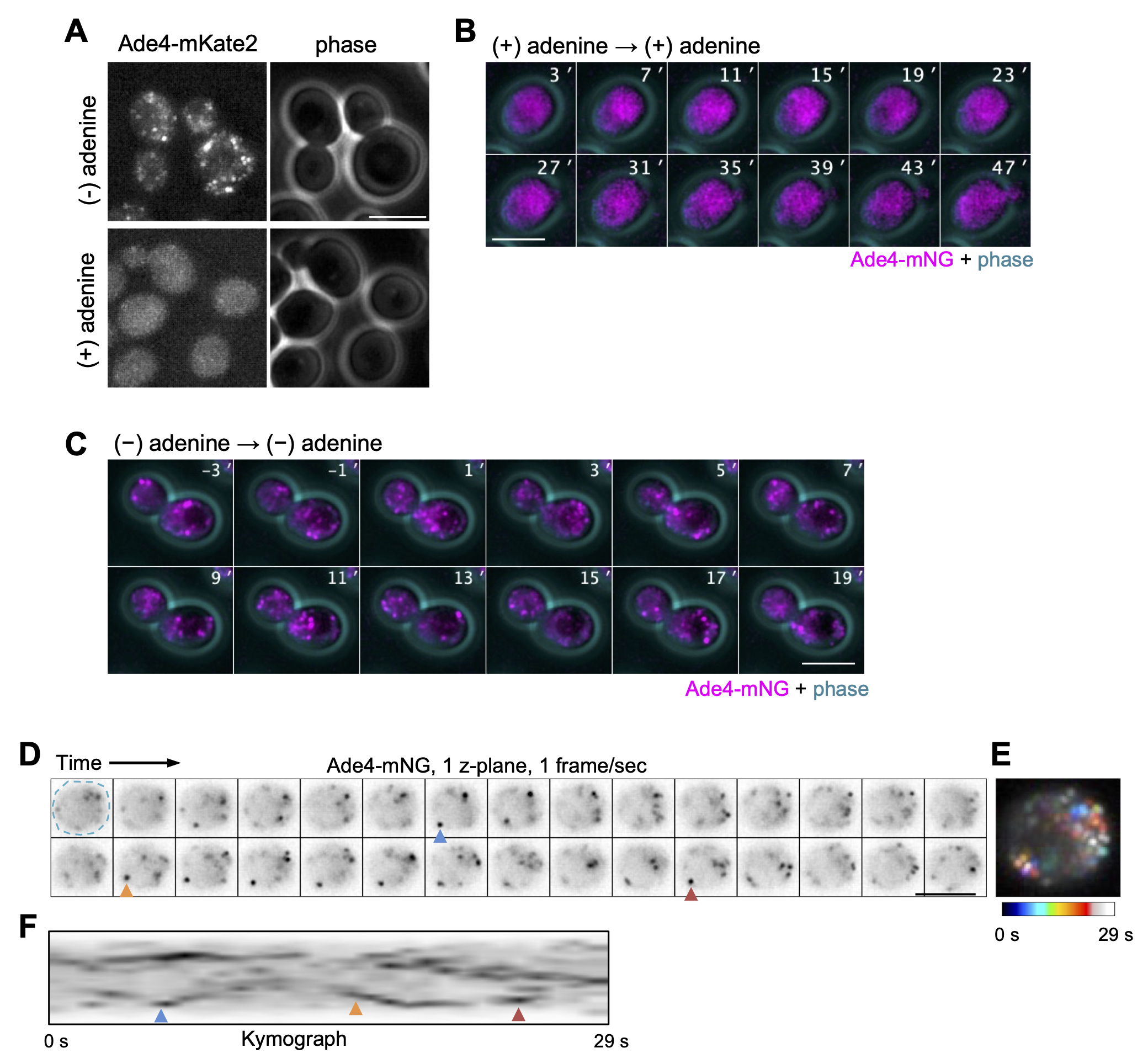

Supplement: S1 Fig — Related to Fig 1. (A) Phase and RFP images of cells expressing Ade4 tagged C-terminally with the red fluorescent protein mKate2. (B) A control experiment of time-lapse imaging of the assembly of Ade4-mNG particles by adenine depletion shown in Fig 1E. Cells were grown in medium containing 0.02-mg/mL adenine and washed with medium containing the same concentration of adenine at t = 0 min. (C) A control experiment of time-lapse imaging of the disassembly of Ade4-mNG particles by adenine supplementation shown in Fig 1F. Cells were grown in the absence of adenine and sterile water was supplemented instead of adenine at t = 0 min. Scale bars = 5 µm. (D–F) Another example of time-lapse imaging of Ade4-mNG particles, related to Fig 1I–1K. Experimental conditions and graph descriptions are the same as in Fig 1I–1K. (TIFF) [file pbio.3003111.s001.tiff]

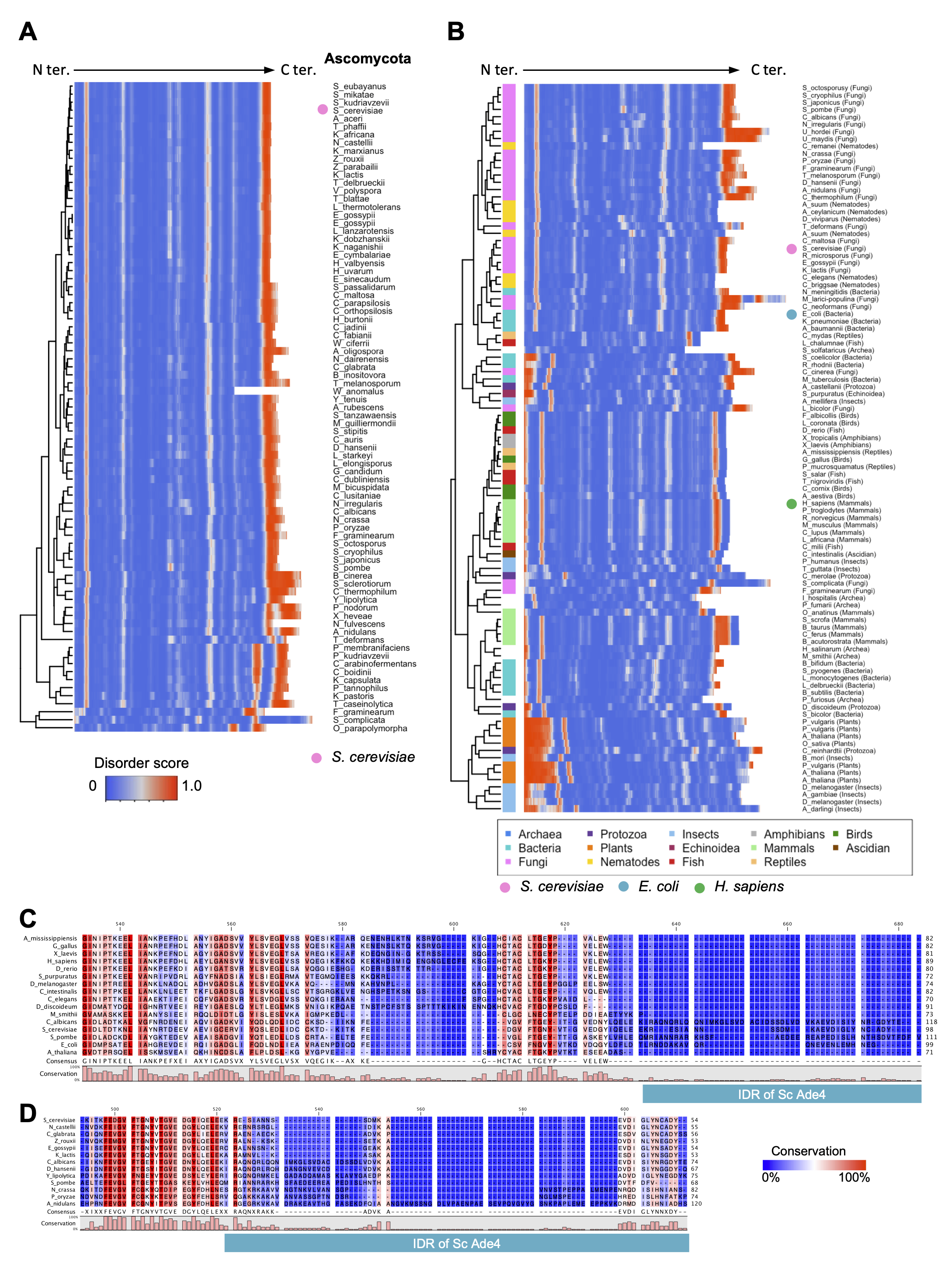

Supplement: S2 Fig — (A) The C-terminal IDR is conserved in PPATs of Ascomycota. Heatmap showing disorder scores for the PPAT amino acid sequences of 81 ascomycota. (B) Heatmap showing disorder scores for the PPAT amino acid sequences of 100 organisms, including 19 ascomycota. Phylogenetic groups are color-coded on the left. (C, D) Amino acid sequences of IDTs vary across species. Multiple amino acid sequence alignments of the C-terminal region of PPAT. In (C), three fungi and 13 representative organisms from the following groups are selected: archaea, bacteria, protozoa, plants, nematodes, insects, echinoidea, fish, amphibians, mammals, reptiles, birds, and ascidians. In (D), 13 species are selected from ascomycota. Green boxes indicate the region corresponding to the C-terminal IDR of Ade4. The data for panels A–B may be found in S1 Data. (TIFF) [file pbio.3003111.s002.tiff]

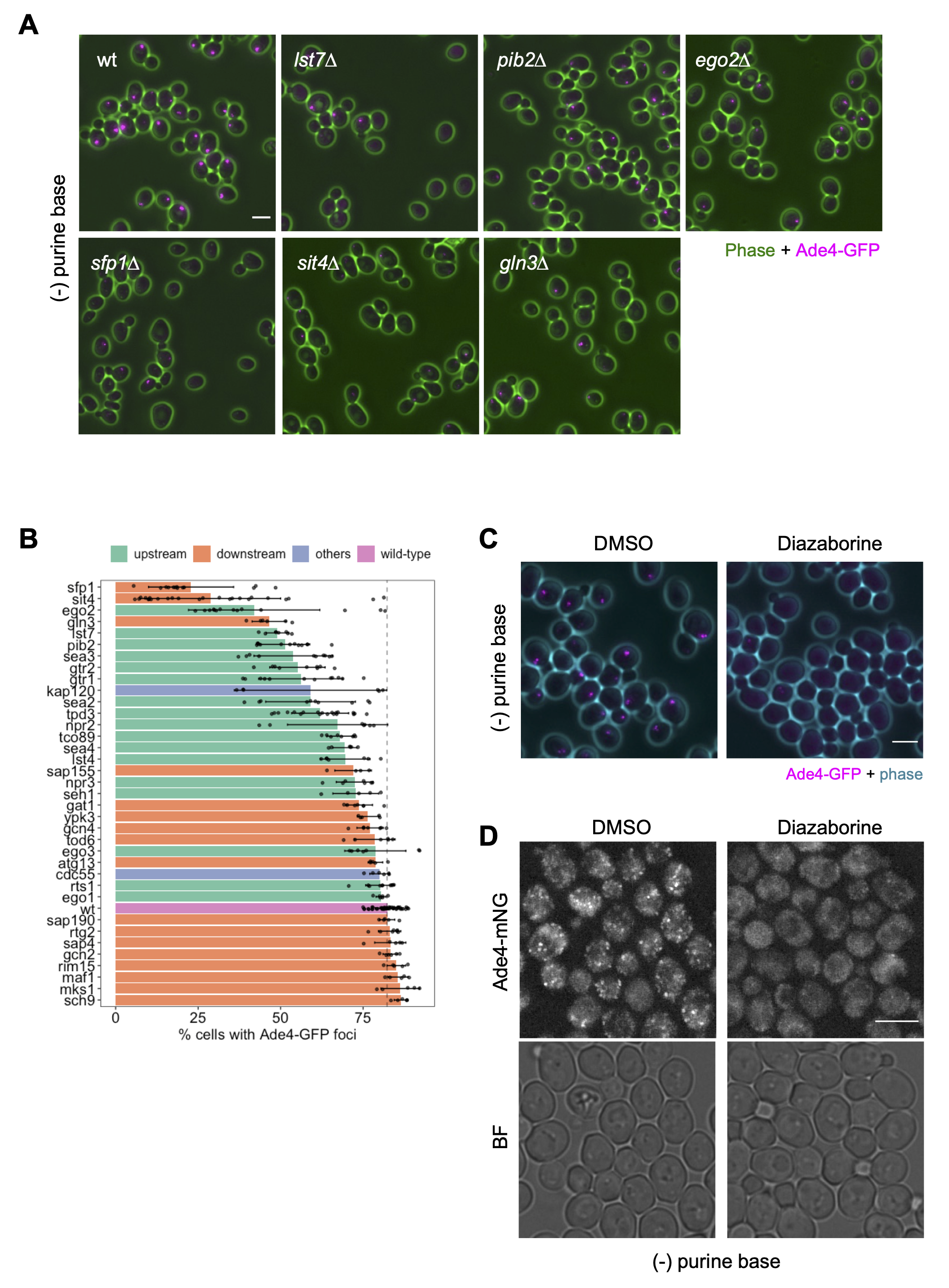

Supplement: S3 Fig — Related to Fig 2. (A) Assembly of Ade4-GFP foci in single gene deletion mutants of the upstream regulator and downstream effector of TORC1 signaling. Cells of the indicated genotype expressing Ade4-GFP were grown in medium containing adenine and then incubated in medium without adenine for 45 min before imaging. (B) Summary of the percentage of cells showing Ade4-GFP foci in the wild type and 37 single-gene deletion mutants. The percentage of cells with Ade4-GFP foci per field of view was plotted. Data were pooled from 2 to 3 independent experiments. Bars and error bars indicate the mean ± 1 SD. (C) The inhibition of ribosome synthesis suppressed the assembly of Ade4-GFP foci. Related to Fig 2J. (D) The inhibition of ribosome synthesis suppressed the assembly of Ade4-mNG condensates. Cells expressing Ade4-mNG were grown in medium containing adenine and then incubated in medium without adenine in the presence of 0.05% (v/v) DMSO or 0.05% DMSO plus 5-µg/mL diazaborine for 45 min before imaging. Scale bars = 5 µm. The data for panel B may be found in S1 Data. (TIFF) [file pbio.3003111.s003.tiff]

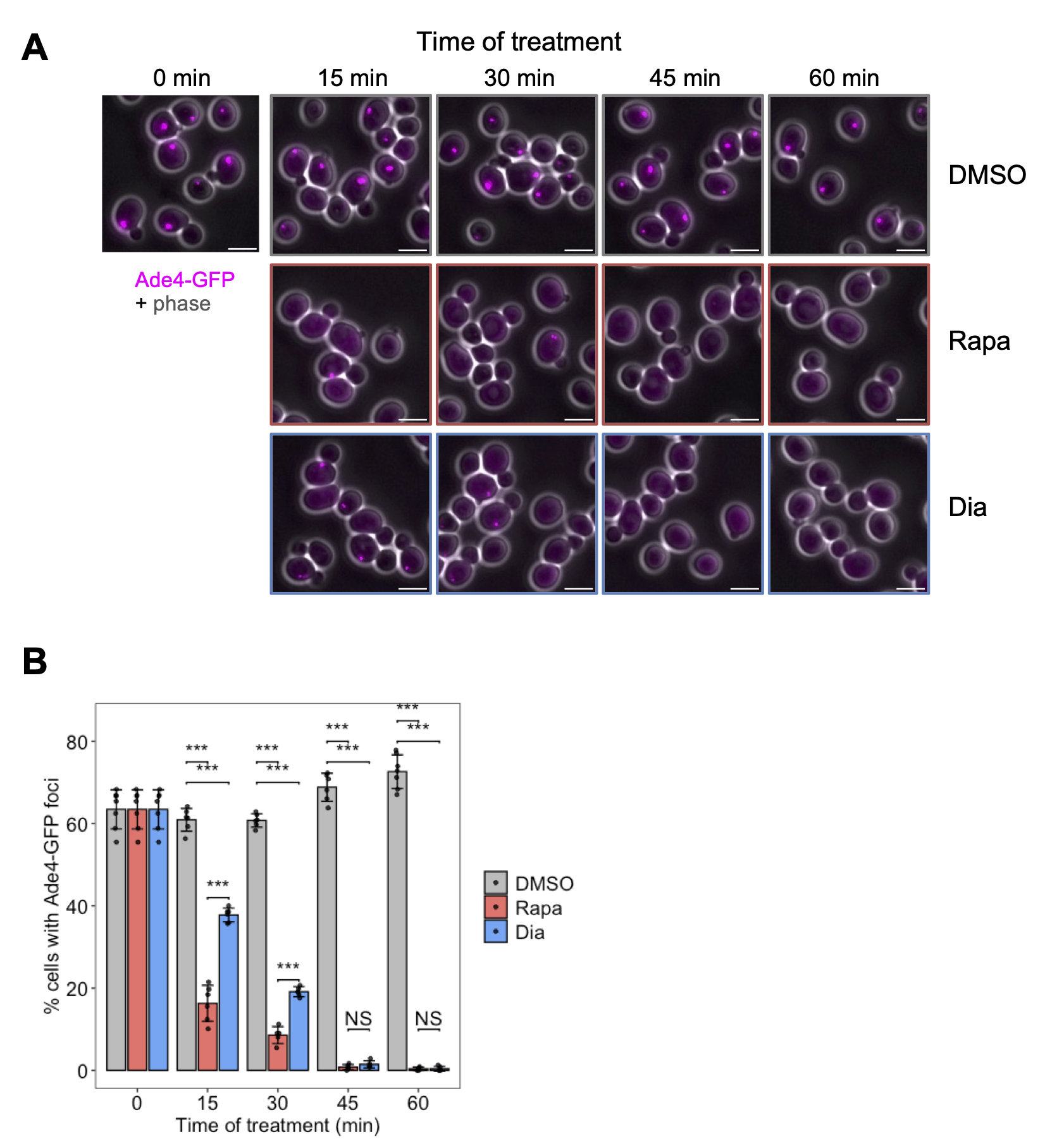

Supplement: S4 Fig — (A) Cells with Ade4-GFP foci in the absence of purine bases were supplemented with 0.02% (v/v) DMSO (DMSO), 0.02% DMSO plus 1-µg/mL rapamycin (Rapa), or 0.02% DMSO plus 5-µg/mL diazaborine (Dia) and then imaged every 15 min for 1 h. Representative cell images are shown. Scale bar = 5 µm. (B) Quantification of the data shown in (A). The percentage of cells with foci per one field of view was quantified and plotted. Bars and e rror bars indicate the mean ± 1SD. More than 5 fields of view were imaged for each condition and ~200 cells were examined per field. Note that data at time = 0 were identical across the three groups because untreated cells were imaged and then divided into three subgroups supplemented with each drug. P-values were calculated using the two-tailed Tukey–Kramer’s multiple comparison test. ***p < 10−4. The data for panel B may be found in S1 Data. (TIFF) [file pbio.3003111.s004.tiff]

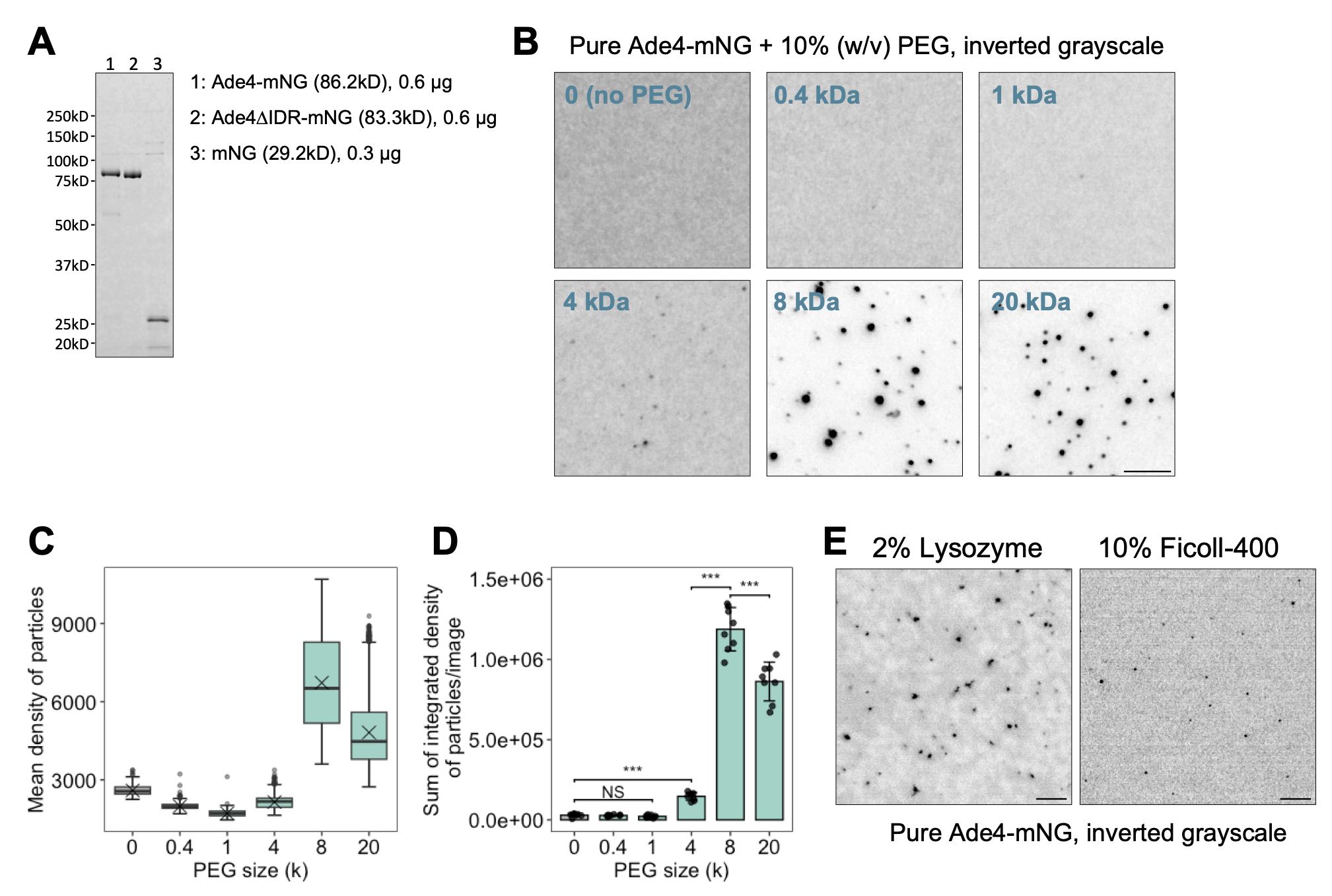

Supplement: S5 Fig — (A) Purified proteins used in the present study. Proteins were resolved by SDS-PAGE and stained with FastGene Q-stain. (B) The in vitro condensation of Ade4-mNG depends on the size of PEG. The supplementation of 0.15-µM Ade4-mNG with 10% PEG of the indicated average molecular weight was performed. (C, D) Quantification of data shown in (B). The mean fluorescence intensities of Ade4-mNG particles were box plotted in (C). Cross marks indicate the population mean. The integrated fluorescence intensities of particles were summed per field and plotted in (D). Bars and error bars indicate the mean ± 1 SD. Seven to 8 fields of view were imaged for each condition, and an average of 399 particles per field were examined. P-values were calculated using the two-tailed Steel–Dwass multiple comparison test. (E) Condensation of Ade4-mNG into particles with crowding agents other than PEG. The supplementation of 0.14-µM Ade4-mNG with the indicated crowding agents was performed. Scale bars = 5 µm. Uncropped gel images are available in S1 Raw images. The data for panels C and D may be found in S1 Data. (TIFF) [file pbio.3003111.s005.tiff]

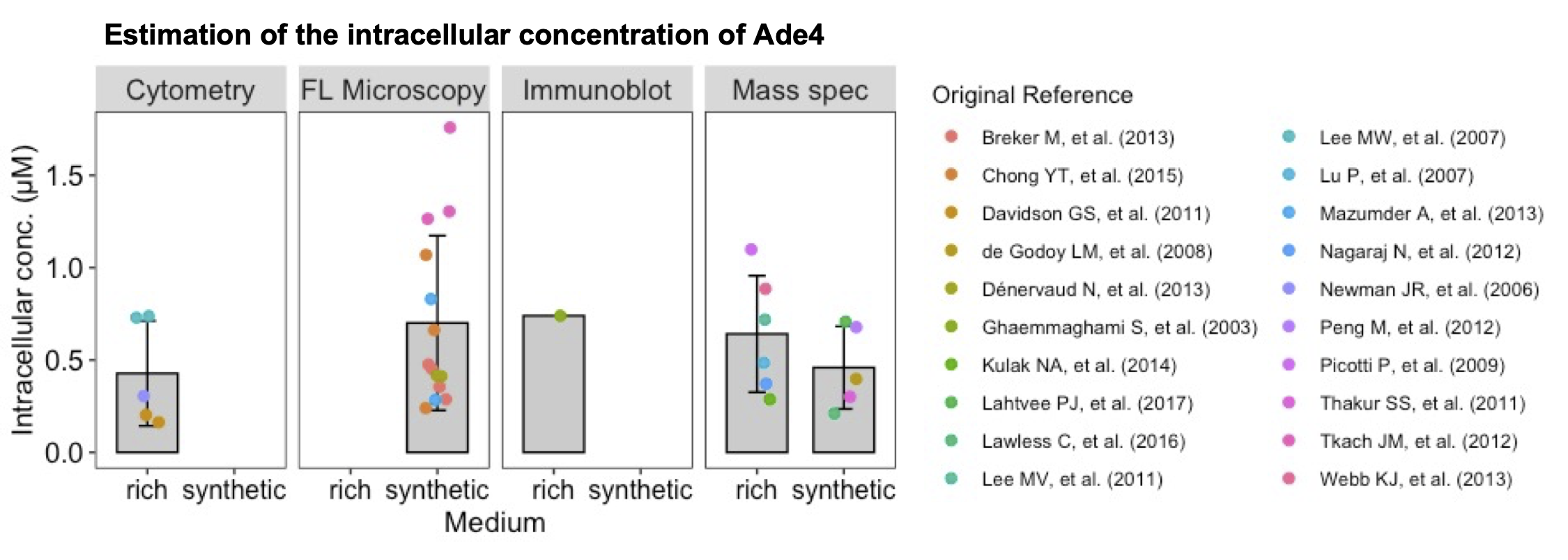

Supplement: S6 Fig — Data regarding the protein abundance of the budding yeast Ade4 were downloaded from the Saccharomyces Genome Database (https://www.yeastgenome.org/locus/S000004915/protein). Data were derived from 20 quantitative genome-wide proteomic studies and normalized to a unit of molecules per cell. These values were converted to micromoles assuming that the average cytoplasmic volume of the haploid yeast cell was 42 fL [71]. Data were classified according to the measuring methods (flow cytometry, quantitative fluorescence microscopy, immunoblotting, and mass spectrometry) and the type of medium (rich or synthetic) and were then plotted. Bars and error bars indicate the mean ± 1 SD. Data may be found in S1 Data. (TIFF) [file pbio.3003111.s006.tiff]

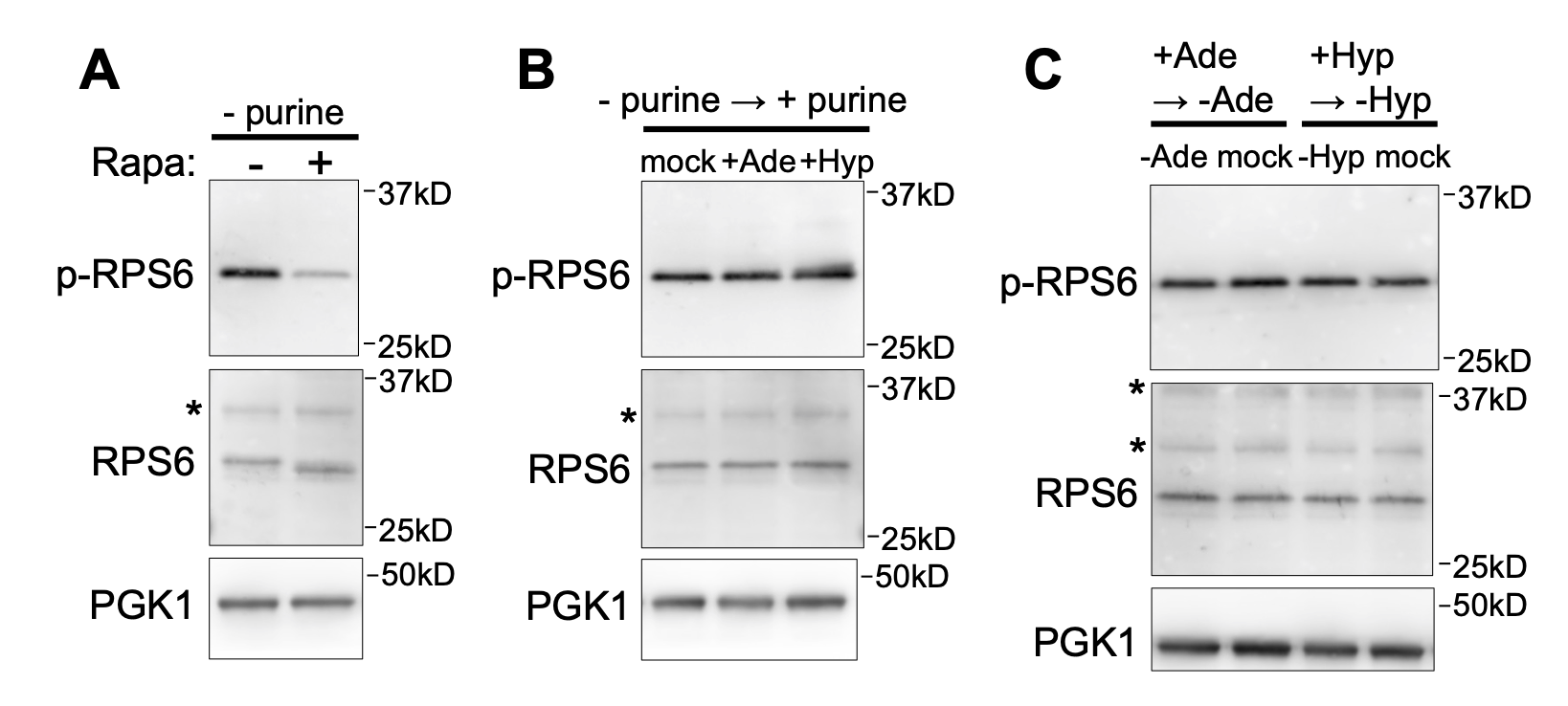

Supplement: S7 Fig — (A) Immunoblot analysis of the phosphorylation state of Rps6. Wild-type (BY4741) cells grown in the absence of purine bases were supplemented with of 0.005% (v/v) DMSO (Rapa: -) or 0.005% DMSO plus 0.5-µg/mL rapamycin (Rapa: +) for 30 min, and total cell extracts were then prepared. Total Rps6 (Rps6) and phosphorylated Rps6 (p-Rps6) were detected as bands at ~30 kDa. The bands of Pgk1 were shown as a loading control. Throughout the figure, asterisks indicate non-specific bands. (B) Supplementation with purine bases did not markedly affect the phosphorylation state of Rps6. Wild-type cells grown in the absence of purine bases were supplemented with 20-µg/mL adenine (+Ade) or 25-µg/mL hypoxanthine (+Hyp) for 30 min, and total cell extracts were prepared. The phosphorylation of Rps6 was analyzed by immunoblots as described in (A). (C) The removal of purine bases did not markedly affect the phosphorylation state of Rps6. Wild-type cells grown in the presence of 20-µg/mL adenine (+Ade) or 25-µg/mL hypoxanthine (+Hyp) were washed three times with the same medium (mock) or the medium lacking purine bases (-Ade, -Hyp), cultured for 40 min, and total cell extracts were then prepared. The phosphorylation of Rps6 was analyzed by immunoblotting as described in (A). Uncropped images of immunoblots are available in S1 Raw images. (TIFF) [file pbio.3003111.s007.tiff]

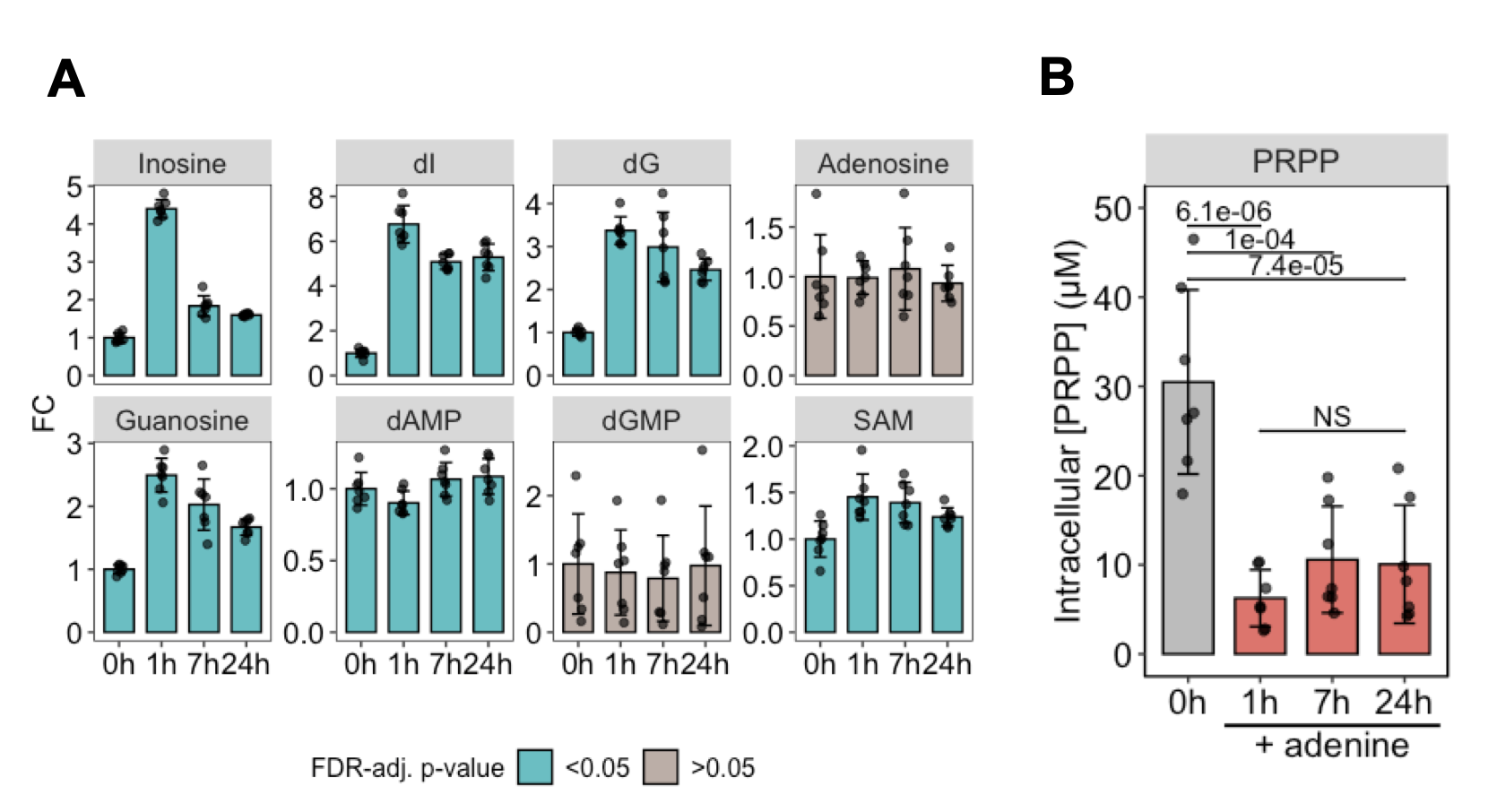

Supplement: S8 Fig — Related to Fig 4. (A) Changes in the indicated metabolites. Experimental conditions and graph descriptions are the same as in Fig 4D. Data may be found in S3 Table. (B) The intracellular concentration of PRPP decreases in the presence of environmental adenine. The amount of PRPP in each sample was converted to an intracellular concentration and plotted. P-values were calculated using the two-tailed Tukey–Kramer’s multiple comparison test. The data for panel B may be found in S1 Data. (TIFF) [file pbio.3003111.s008.tiff]

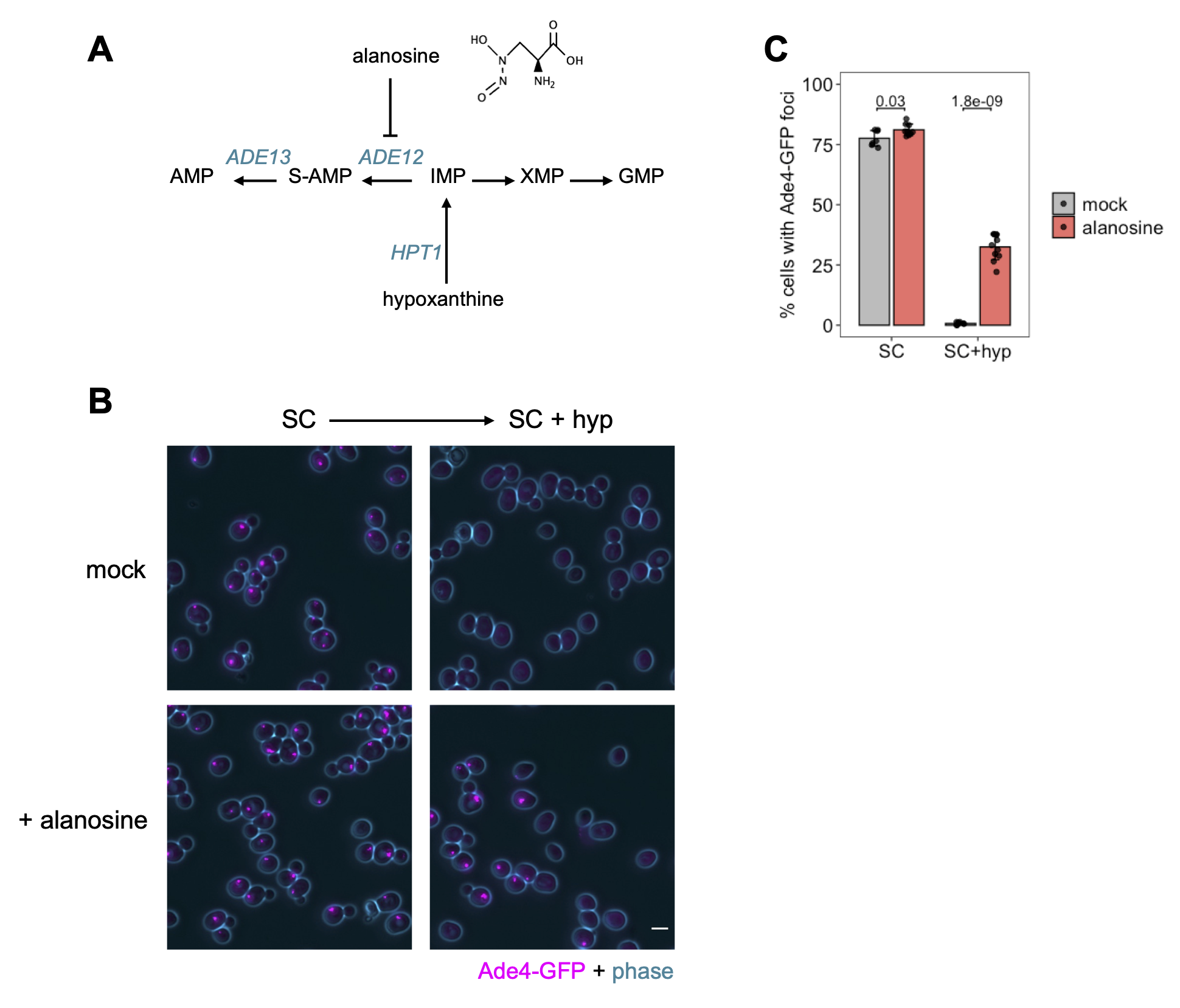

Supplement: S9 Fig — Related to Fig 5. (A) Alanosine inhibits the adenylosuccinate synthetase Ade12. (B) Assembly of Ade4-GFP foci in the presence of alanosine. Cells expressing Ade4-GFP were incubated in medium lacking adenine in the presence of 0 (mock) or 30 µg/mL alanosine (+alanosine) for 40 min to allow for the assembly of Ade4-GFP foci and were then imaged (SC). Cells were further treated with the same medium supplemented with 24-µg/mL hypoxanthine for 15 min and were then imaged again (SC + hyp). Scale bar = 5 µm. (C) Quantification of data shown in (B). The percentage of cells with foci per field of view (containing 182 ± 46 cells) was quantified and plotted. Data were pooled from two independent experiments. Bars and error bars indicate the mean ± 1 SD. The significance of differences was tested using the unpaired two-tailed Welch’s t test. P-values vs. the mock are shown. The data for panel C may be found in S1 Data. (TIFF) [file pbio.3003111.s009.tiff]

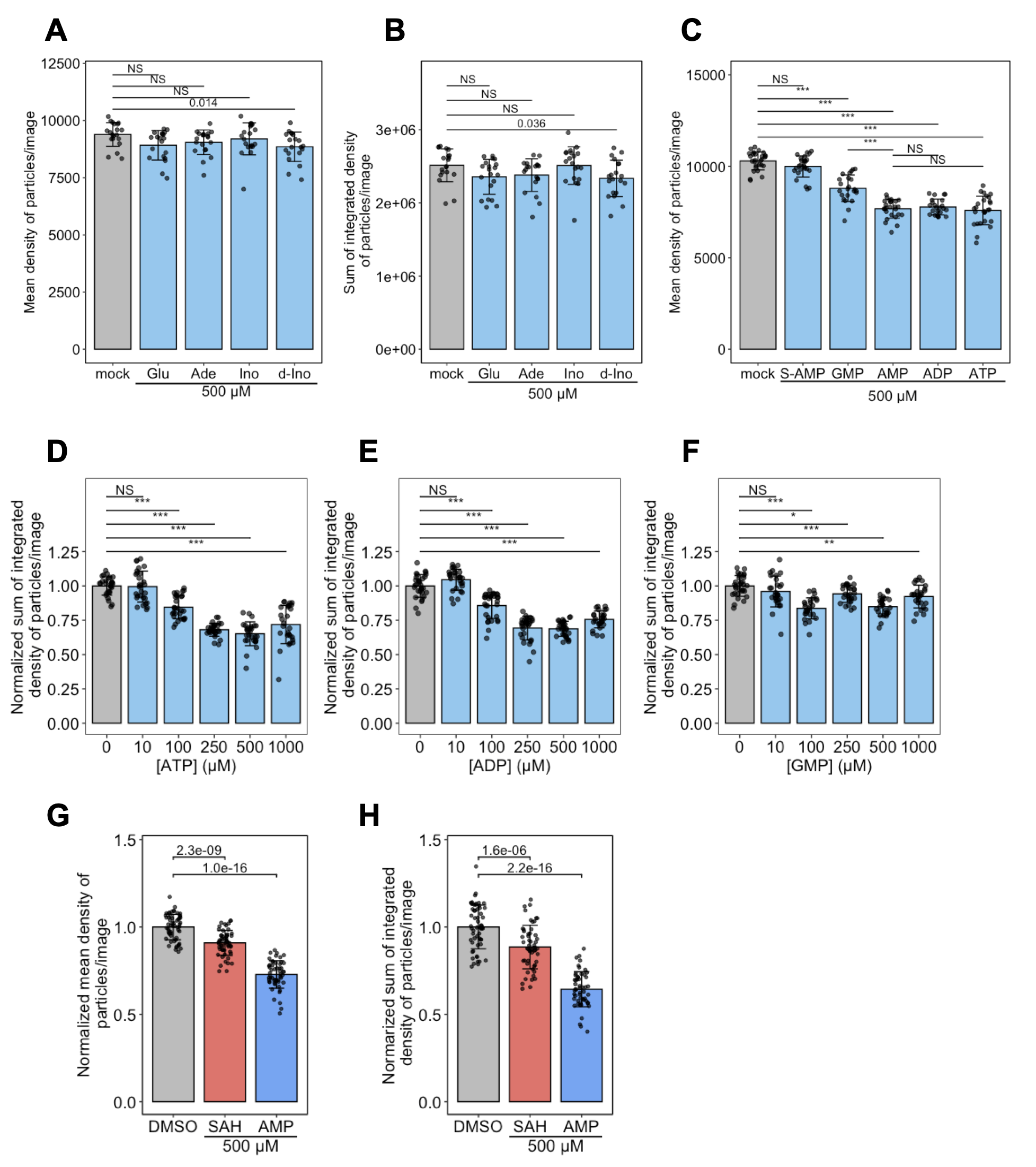

Supplement: S10 Fig — (A, B) Effects of purine base derivatives on the in vitro assembly of Ade4 condensates. The supplementation of 0.2-µM Ade4-mNG with 10% PEG8K was performed in the presence of the indicated compound. Glucose (Glu) was used as a negative control. The integrated fluorescence intensities of condensates were summed per field and plotted in (A). The mean fluorescence intensities of Ade4-mNG condensates were averaged per field and plotted in (B). Bars and error bars indicate the mean ± 1 SD. Twenty fields of view were imaged for each condition from two independent preparations, and an average of 1,452 particles per field were examined. P-values were calculated using the two-tailed Steel’s multiple comparison test. Glu, glucose; Ade, adenine; Ino, inosine; d-Ino, deoxy-inosine. (C) Effects of purine nucleotides on the in vitro assembly of Ade4 condensates. Data were from the same experiment shown in Fig 6A. The mean fluorescence intensities of Ade4-mNG condensates were averaged per field and plotted. Bars and error bars indicate the mean ± 1 SD. P-values were calculated using the two-tailed Tukey–Kramer’s multiple comparison test. (D–F) The supplementation of 0.2-µM Ade4-mNG with 10% PEG8K was performed in the presence of the indicated concentrations of ADP (D), ATP (E), and GMP (F). The integrated fluorescence intensities of the condensates were summed per field, normalized to the mean at 0 µM, and plotted. Bars and error bars indicate the mean ± 1 SD. From two independent preparations, 25–31 fields of view were imaged for each condition, and an average of 1,200 particles per field were examined. P-values were calculated using the two-tailed Steel’s multiple comparison test. (G, H) Inhibitory effect of SAH on the in vitro condensation of Ade4-mNG. The supplementation of 0.2-µM Ade4-mNG with 10% PEG8K was performed in the presence of 0.5% (v/v) DMSO and 500-µM SAH or AMP and imaged. The average fluorescence intensities (G) and integrated fluorescence intensities (H) of the [file pbio.3003111.s010.tiff]

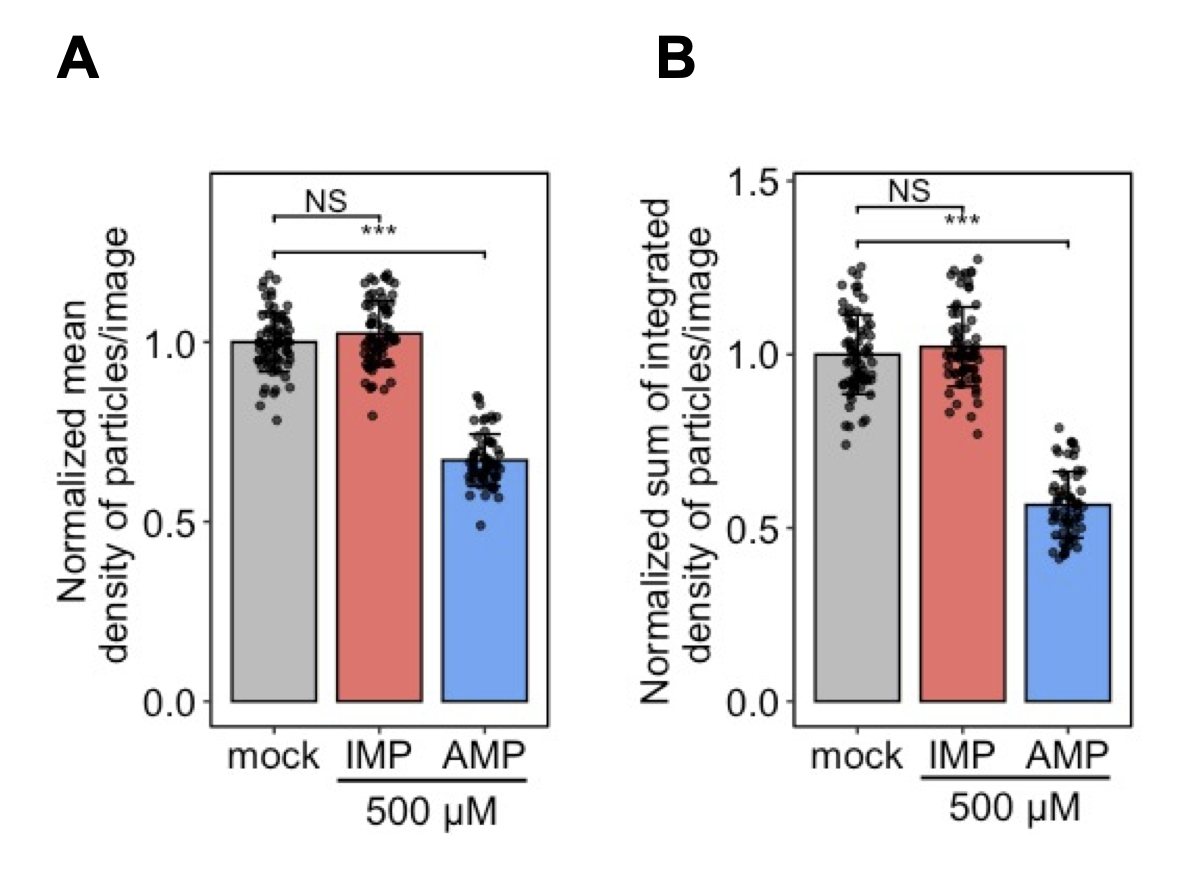

Supplement: S11 Fig — (A, B) IMP did not affect the in vitro condensation of Ade4-mNG. The supplementation of 0.2-µM Ade4-mNG with 10% PEG8K was performed in the presence of 500 µM IMP or AMP. The average fluorescence intensities (A) and integrated fluorescence intensities (B) of the condensates summed per field were normalized to the mean of the mock and plotted. Bars and error bars indicate the mean ± 1 SD. From 4 independent preparations, 62–72 fields of view were imaged for each condition. P-values were calculated using the two-tailed Steel’s multiple comparison test. The data for panels A and B may be found in S1 Data. (TIFF) [file pbio.3003111.s011.tiff]

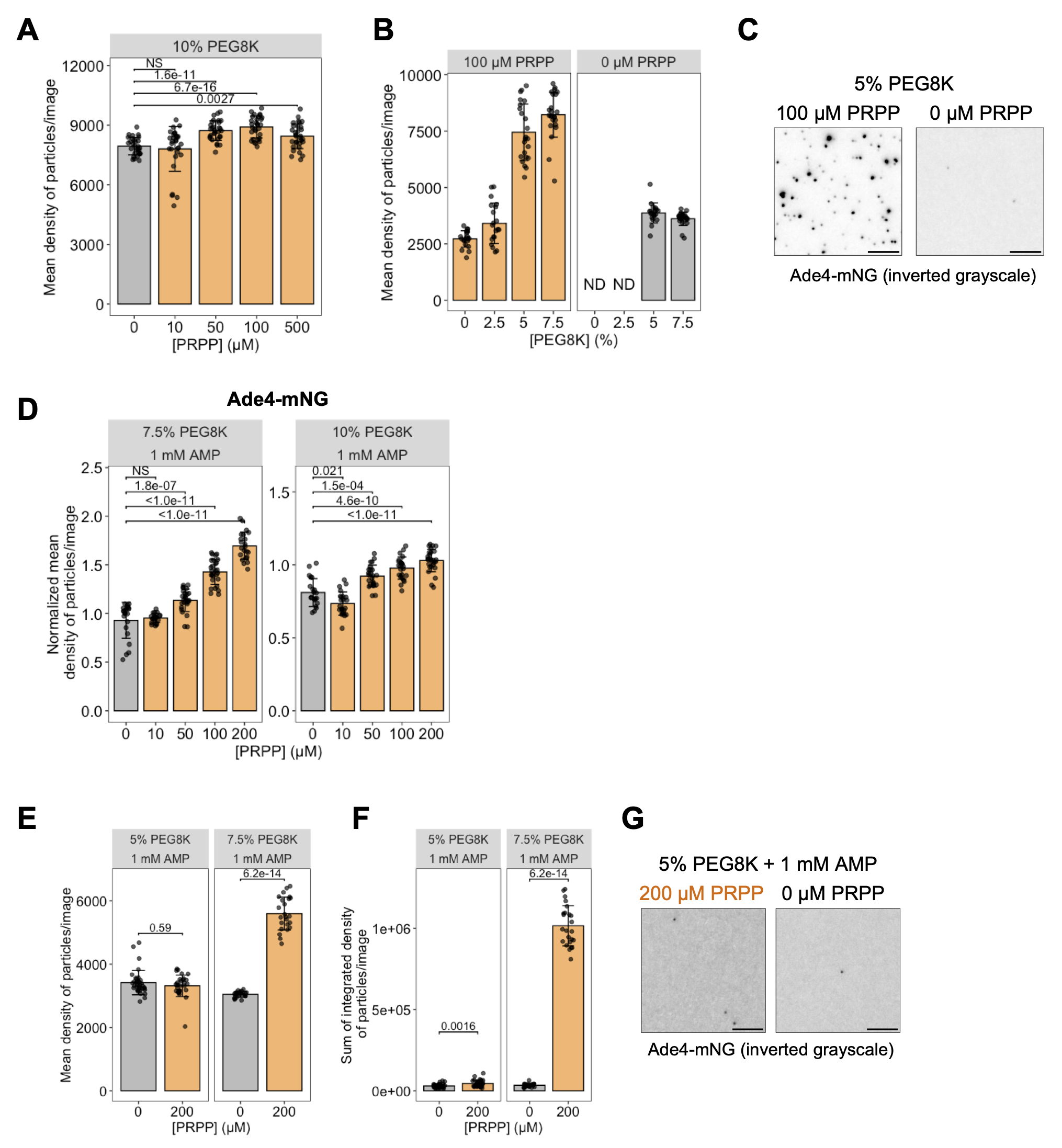

Supplement: S12 Fig — (A) PRPP augments the in vitro assembly of Ade4 condensates. Data were from the same experiment shown in Fig 7A. The mean fluorescence intensities of Ade4-mNG condensates were averaged per field and plotted. Throughout the figure, bars and error bars indicate the mean ± 1 SD. P-values were calculated using the two-tailed Steel’s multiple comparison test. (B, C) PRPP promotes the formation of Ade4 condensates in vitro at suboptimal PEG concentrations. Data were from the same experiment shown in Fig 7B and 7C. The mean fluorescence intensities of Ade4-mNG condensates were averaged per field and plotted in (B). ND, not determined. Representative images at 5% PEG are shown in (C). (D) PRPP antagonizes the inhibitory effects of AMP. Data were from the same experiment shown in Fig 7D and 7E. The mean fluorescence intensities of Ade4-mNG condensates were averaged per field, normalized to the mean of the mock control (no AMP and no PRPP), and plotted. P-values were calculated using the two-tailed Steel’s multiple comparison test. (E–G) In the presence of 1-mM AMP and the indicated concentrations of PRPP, 0.2-µM Ade4-mNG was supplemented with 5% or 7.5% PEG8K. From 2 to 3 independent preparations, 24–36 fields of view were imaged for each condition. (E) The mean fluorescence intensities of Ade4-mNG condensates were averaged per field and plotted. Throughout the figure, bars and error bars indicate the mean ± 1 SD. (F) The integrated fluorescence intensities of the condensates were summed per field and plotted. P-values were calculated using the two-tailed Mann–Whitney U test. Representative images under 5% PEG conditions are shown in (G). Scale bars = 5 µm. Note that although PRPP increased the number of extremely small condensates, it failed to strongly promote the condensation of Ade4-mNG. The data for panels A, B, and D–F may be found in S1 Data. (TIFF) [file pbio.3003111.s012.tiff]

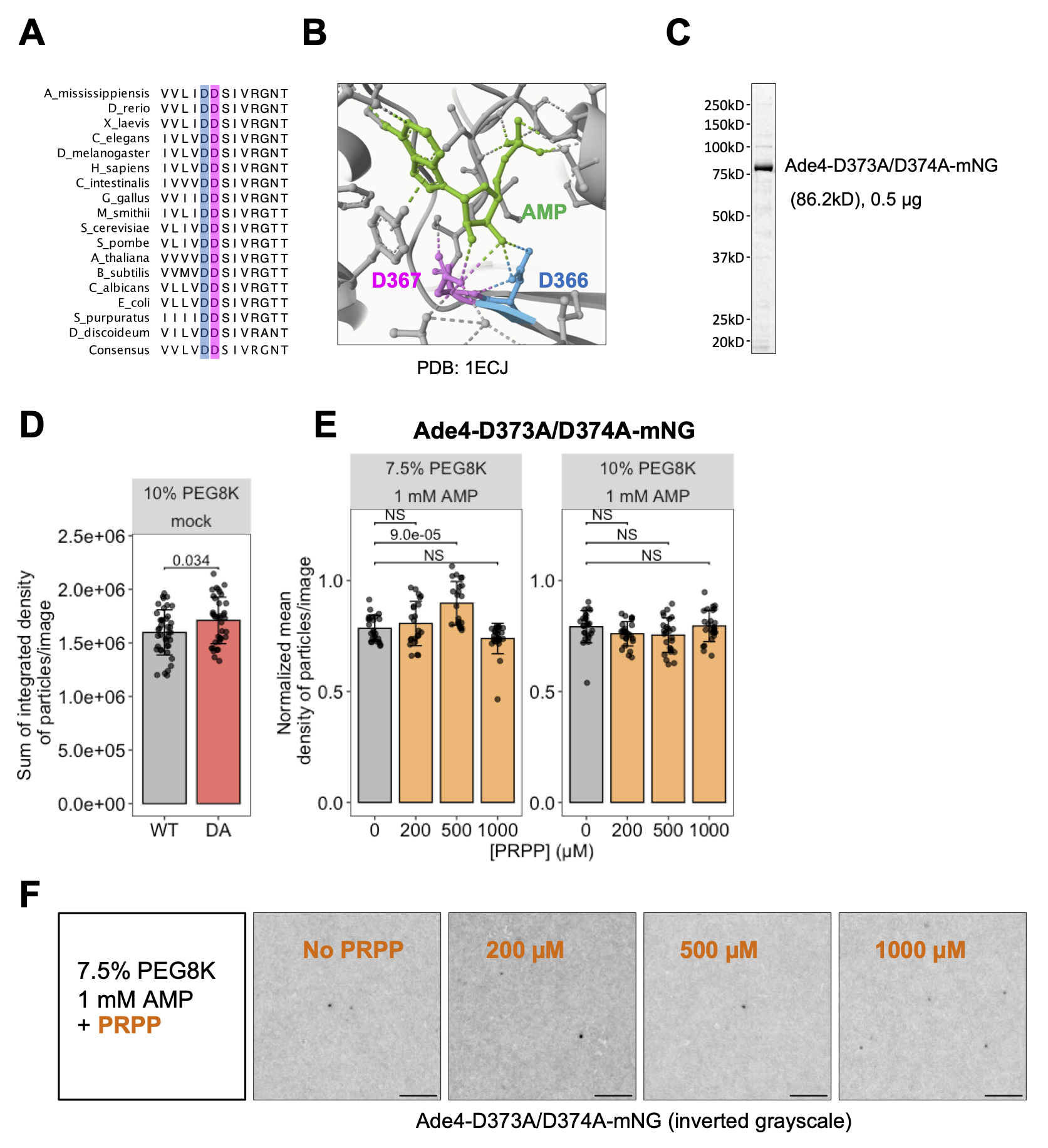

Supplement: S13 Fig — (A) The amino acid residues of the PRPP binding motif in PPAT are well conserved among species. (B) Interactions between an AMP and specific amino acid residues in the PRPP binding motif in E. coli PPAT (pdb file: 1ecj). The dotted line indicates a hydrogen bond. (C) The purified Ade4-D373A/D374A-mNG (Ade4-DA-mNG) protein. Proteins were resolved by SDS-PAGE and stained with FastGene Q-stain. (D) Ade4-DA-mNG condensated into particles under molecular crowding conditions. The supplementation of 0.2-µM Ade4-mNG (WT) and Ade4-DA-mNG (DA) with 10% PEG8K was performed and imaged. The integrated fluorescence intensities of the condensates were summed per field and plotted. From 2 independent preparations, >36 fields of view were imaged for each condition. P-values were calculated using the two-tailed Mann–Whitney U test. (E, F) PRPP did not antagonize AMP in condensate formation in the DA mutant. Data were from the same experiment shown in Fig 7K. The mean fluorescence intensities of Ade4-D373A/D374A-mNG condensates were averaged per field, normalized to the mean of the mock control (no AMP or PRPP), and plotted in (E). P-values were calculated using the two-tailed Steel’s multiple comparison test. Representative images at 7.5% PEG are shown in (F). Scale bars = 5 µm. Uncropped gel images are available in S1 Raw images. The data for panels D and E may be found in S1 Data. (TIFF) [file pbio.3003111.s013.tiff]

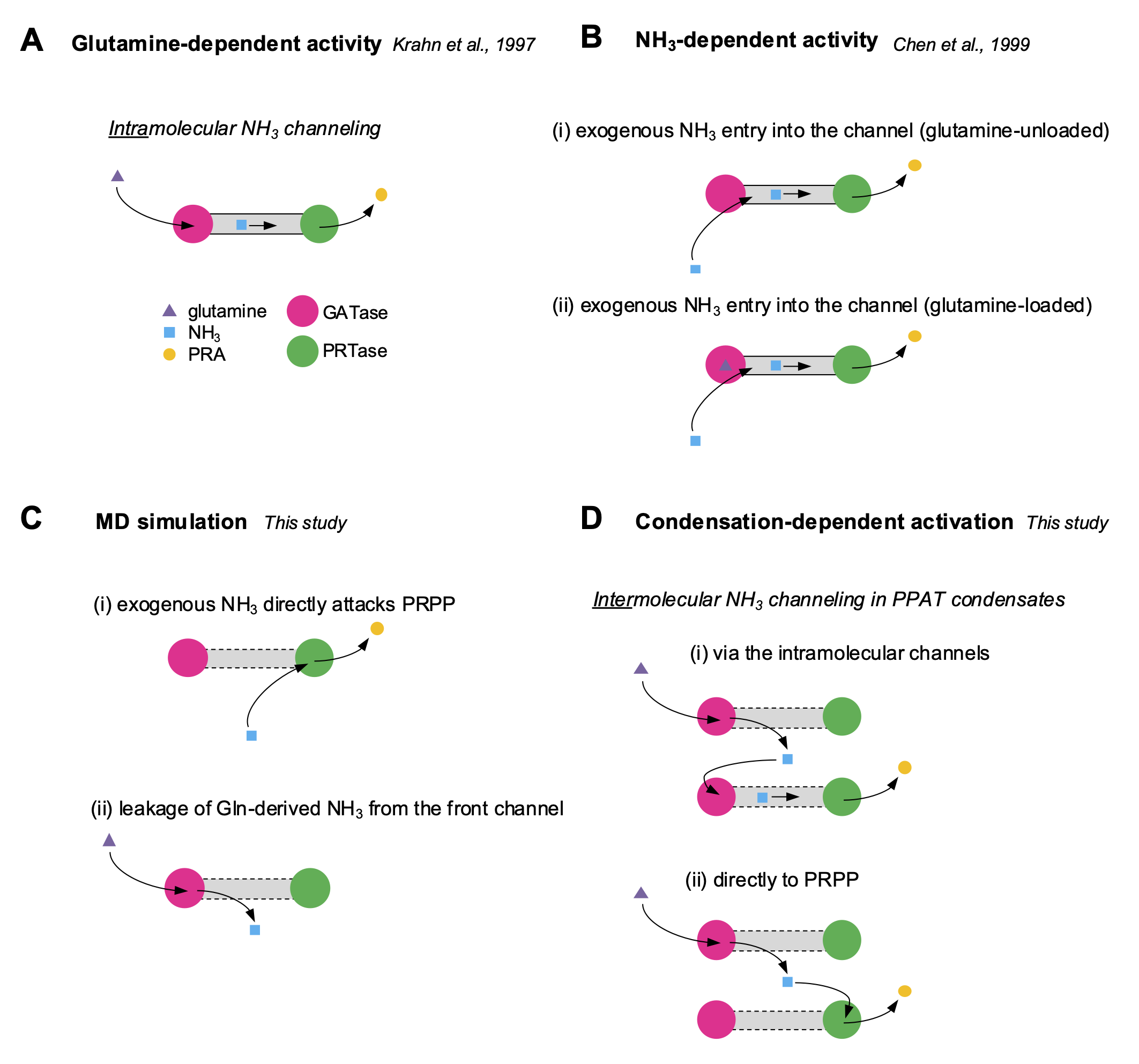

Supplement: S14 Fig — (A) Intramolecular channeling of glutamine-derived NH3 proposed by Krahn and colleagues [35]. (B) Mechanism of NH3-dependent activity proposed by Chen and colleagues [54]. External NH3 enters the channel from the glutamine site irrespective of glutamine binding. (C) The MD simulation in the present study suggests that glutamine-derived NH3 leaks through the front channel following the attack of exogenous NH3. (D) Condensation-dependent activation of Ade4 proposed by the present study. Glutamine-derived NH3 leaked from the front channel rapidly diffuses away. In the Ade4 condensate, the opportunity for NH3 to be captured by other molecules and further reacted with PRPP will increase. This activation may be described as intermolecular NH3 channeling. PRPP is not depicted in the schema. (TIFF) [file pbio.3003111.s014.tiff]

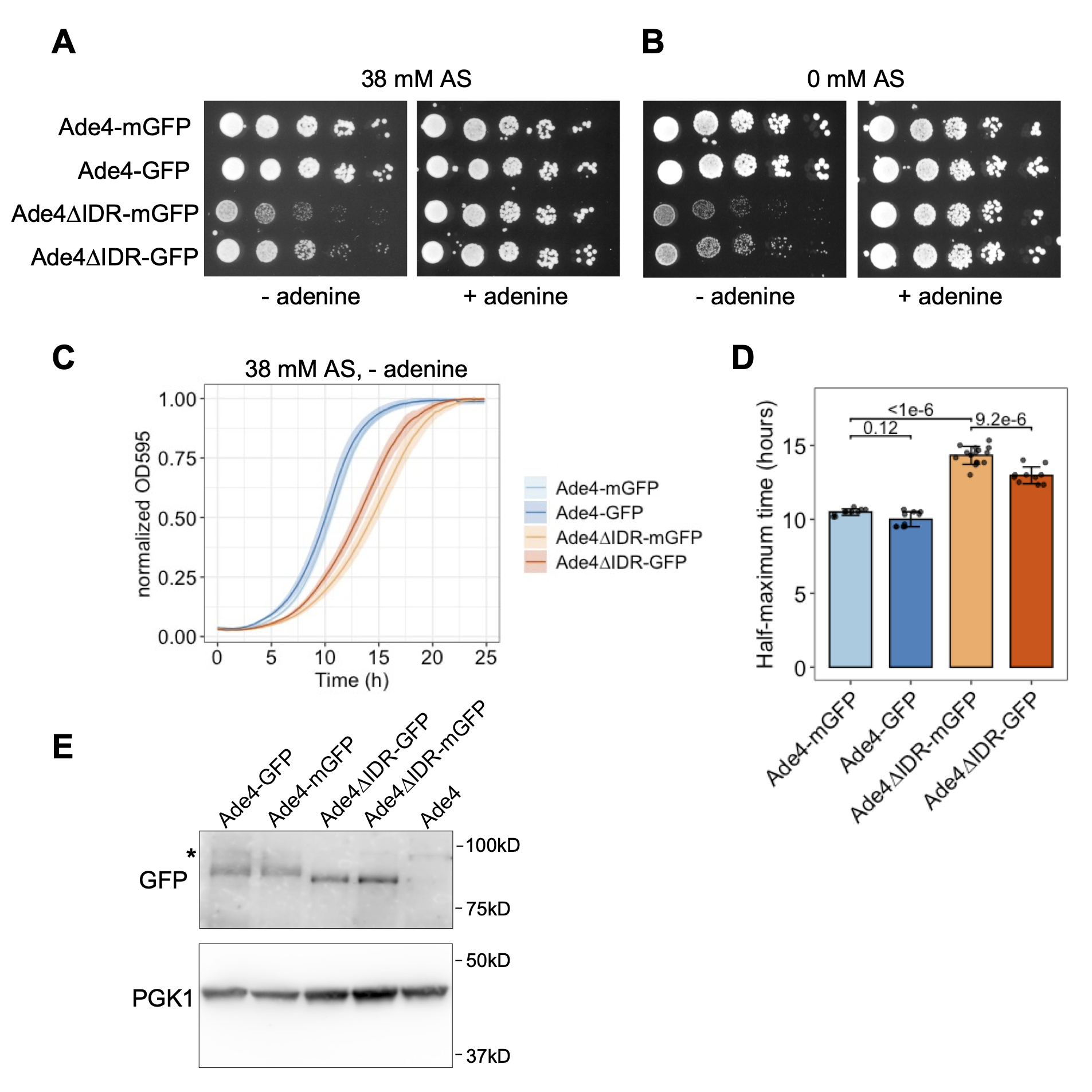

Supplement: S15 Fig — (A, B) Growth of cells expressing Ade4-mGFP, Ade4-GFP, Ade4∆IDR-mGFP, and Ade4∆IDR-GFP on solid media. Cells of each strain were serially diluted (5-fold), spotted on 2% glucose medium containing 38 mM AS (A) and 8 mM proline (no AS) (B), and then grown at 30°C for 2 days. (C) Growth curves of cells expressing Ade4-mGFP, Ade4-GFP, Ade4∆IDR-mGFP, and Ade4∆IDR-GFP in liquid media without adenine. Cells were grown at 30°C for 25 h in medium containing the indicated nitrogen source in the absence of adenine. Data are the mean of 10–15 replicates, and the shaded area indicates ±1 SD. (D) Quantification of the data shown in (C). The time to half-maximum density (corresponding to normalized OD595 = 0.5) was calculated and plotted. Bars and error bars indicate the mean ± 1 SD. P-values were calculated using the Tukey–Kramer multiple comparison test. (E) Immunoblot analysis of the protein level of Ade4 tagged C-terminally with GFP or mGFP. Cells expressing the indicated Ade4 construct were grown to the mid-log phase in the absence of adenine, supplemented with 0.02-mg/mL adenine, cultured for another 20 min to dissolve Ade4 condensation, and total cell extracts were then prepared. Ade4 proteins were detected as bands at ~85 kDa by an anti-GFP antibody. Asterisks indicate non-specific bands. The bands of PGK1 were shown as a loading control. Uncropped images of immunoblots are available in S1 Raw images. The data for panels C and D may be found in S1 Data. (TIFF) [file pbio.3003111.s015.tiff]

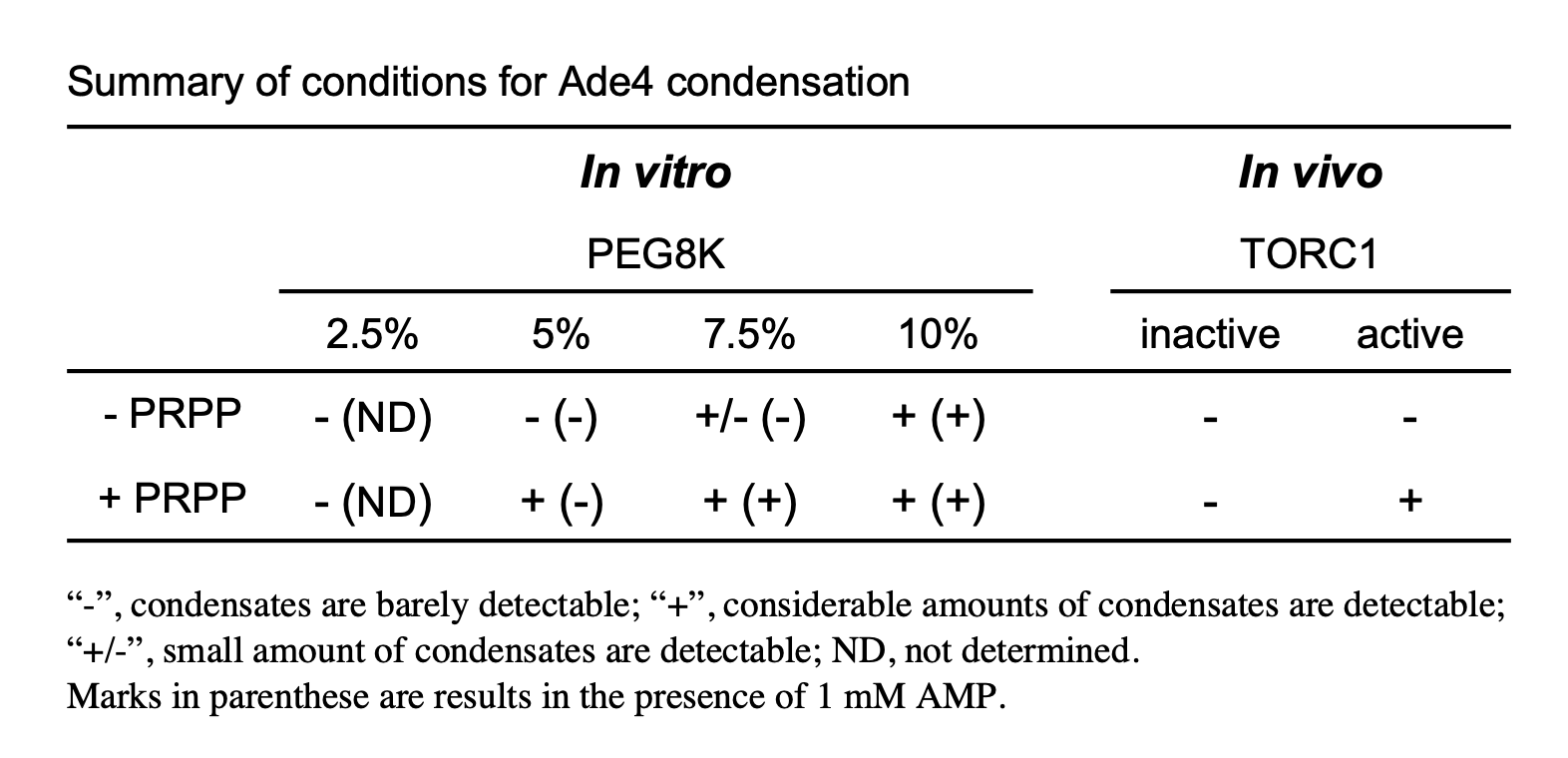

Supplement: S16 Fig — In vivo results are based on data shown in Figs 1, 2, and 4. In vitro results are based on data shown in Figs 3C, 6C, 7A–B, 7D, and S12F. (TIFF) [file pbio.3003111.s016.tiff]
