# Supplementary material for: Phase separation of the PRPP amidotransferase into dynamic condensates promotes de novo purine synthesis in yeast
Source: PLoS Biol. 2025 Apr 10;23(4):e3003111. doi: 10.1371/journal.pbio.3003111 (PMC12017579; doi:10.1371/journal.pbio.3003111)
Supplement: S1 Raw Images — Each figure panel displays uncropped, minimally adjusted images of immunoblots or stained gels with annotations of experimental samples and molecular weights. The cropped area is indicated by a dotted rectangle. Lanes not included in the final figure are marked with an “X” above the lane. (PDF) [file pbio.3003111.s023.pdf]

Uncropped images of immunoblots and gel shown in:

Phase separation of the PRPP amidotransferase into dynamic condensates promotes de novo purine synthesis in yeast

by Masak Takaine, Rikuri Morita, Yuto Yoshinari, and Takashi Nishimura

Each figure panel displays uncropped, minimally adjusted images of immunoblot or stained gel with annotations of experimental samples and molecular weights. The cropped area is indicated by a dotted rectangle. Lanes not included in the final figure are marked with an “X” above the lane.

Fig 9B

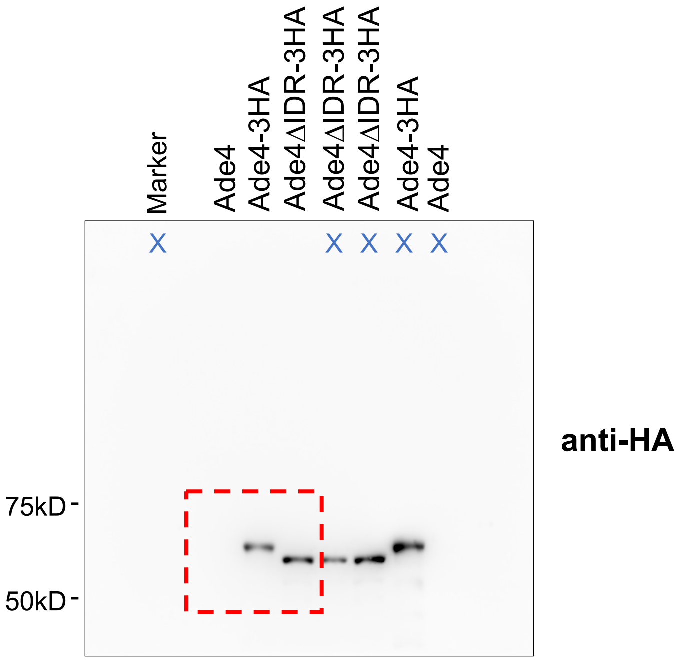

Marker
Ade4
Ade4-3HA
Ade4ΔIDR-3HA
Ade4ΔIDR-3HA
Ade4ΔIDR-3HA
Ade4-3HA
Ade4
X
X X X X
75kD
50kD
anti-HA

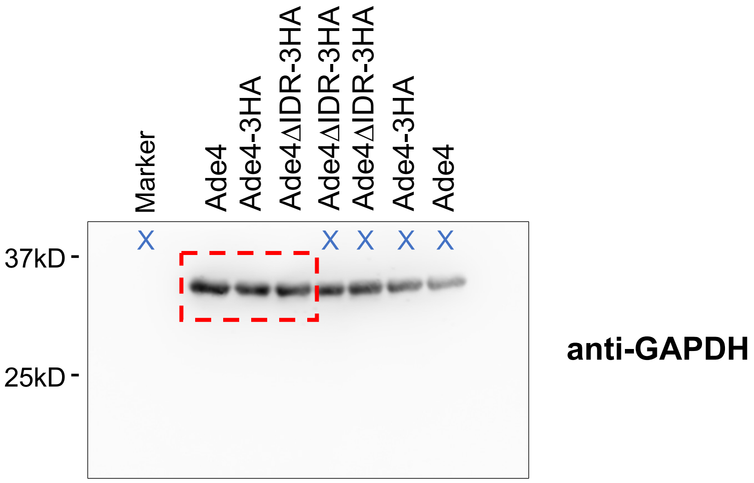

Marker
Ade4
Ade4-3HA
Ade4ΔIDR-3HA
Ade4ΔIDR-3HA
Ade4ΔIDR-3HA
Ade4-3HA
Ade4
X
X X X X
37kD
25kD
anti-GAPDH

Fig S5A

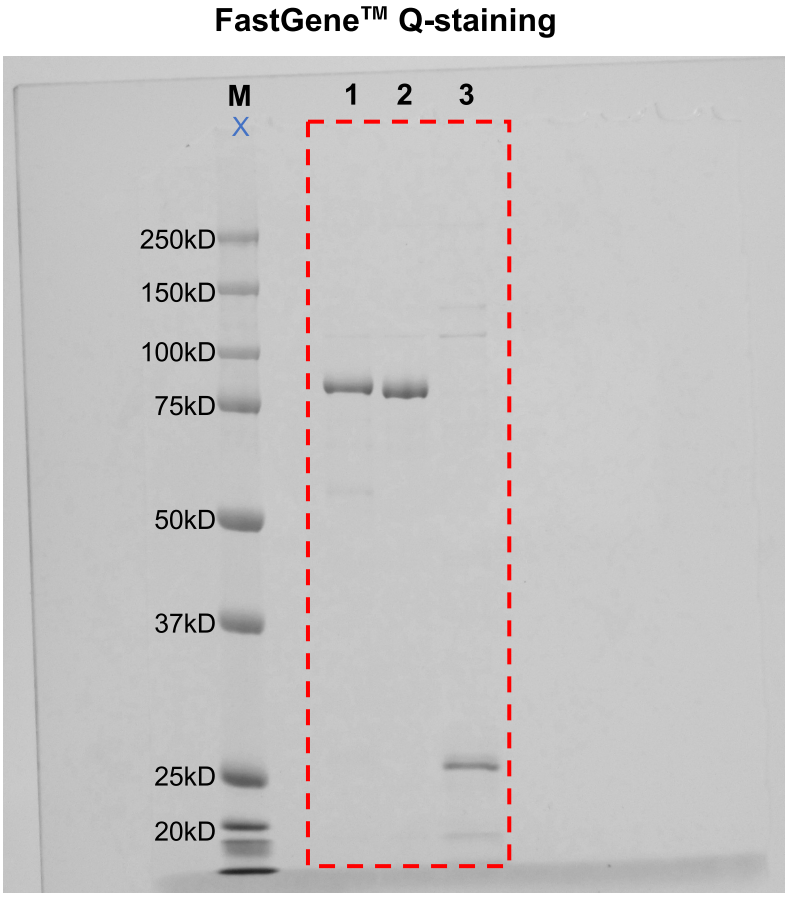

M: Precision plus protein dual color standards (Bio-Rad)

1: Ade4-mNG (86.2kD), 0.6 μg

2: Ade4ΔIDR-mNG (83.3kD), 0.6 μg

3: mNG (29.2kD), 0.3 μg

S7A Fig

S7B Fig

*, a non-specific band in cell extract.

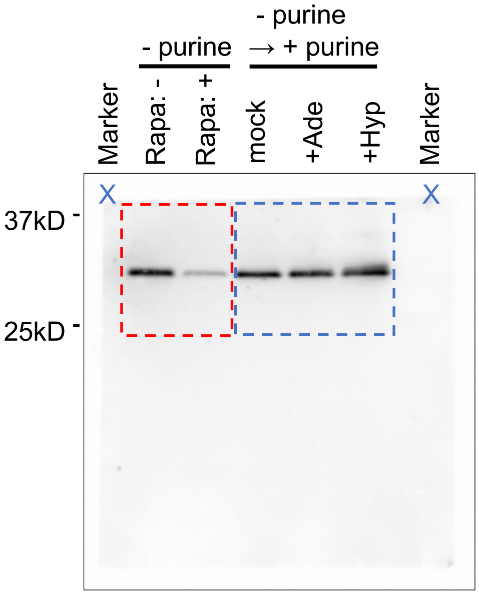

anti-p-RPS6

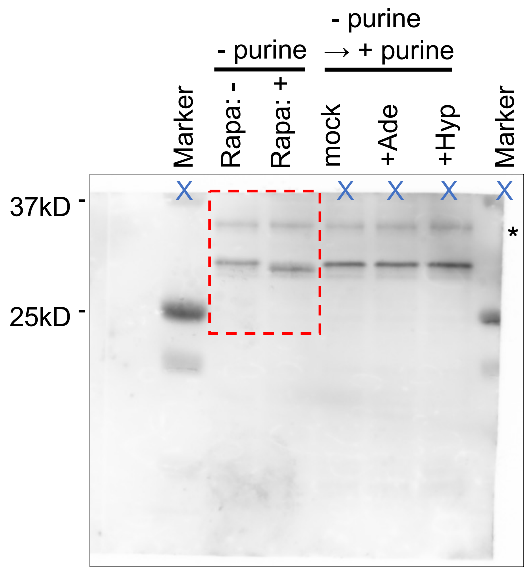

anti-RPS6 (10 min exposure)

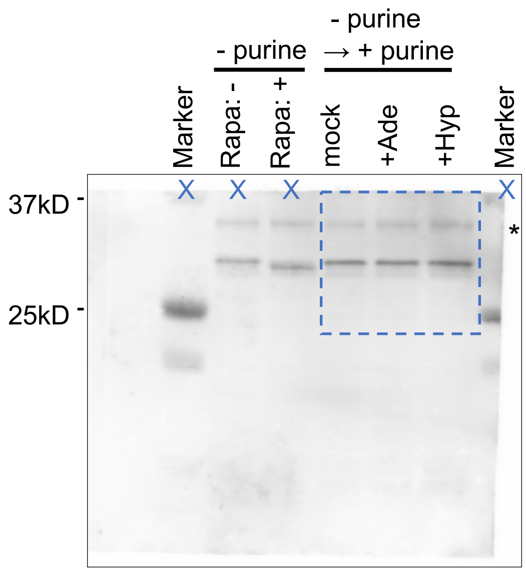

anti-RPS6 (8 min exposure)

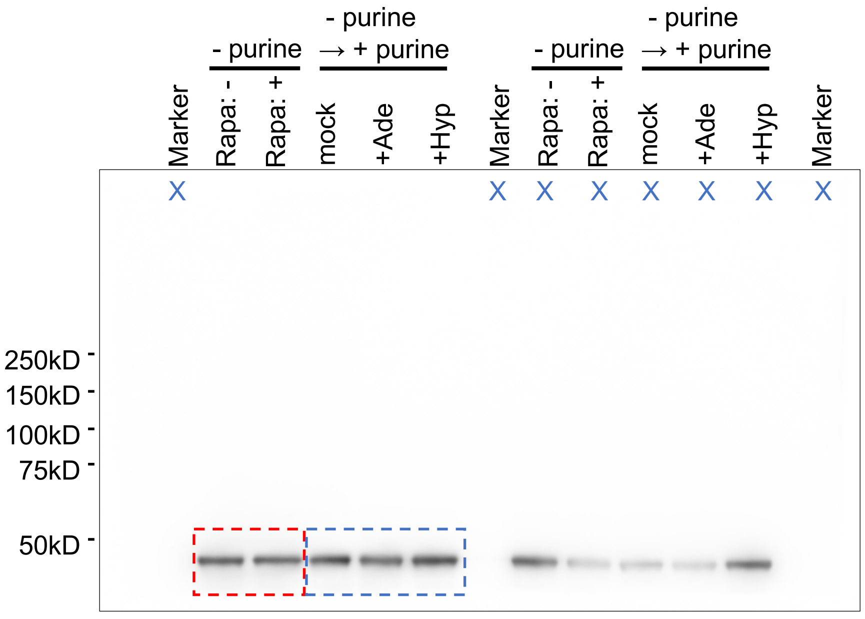

anti-PGK1

S7C Fig

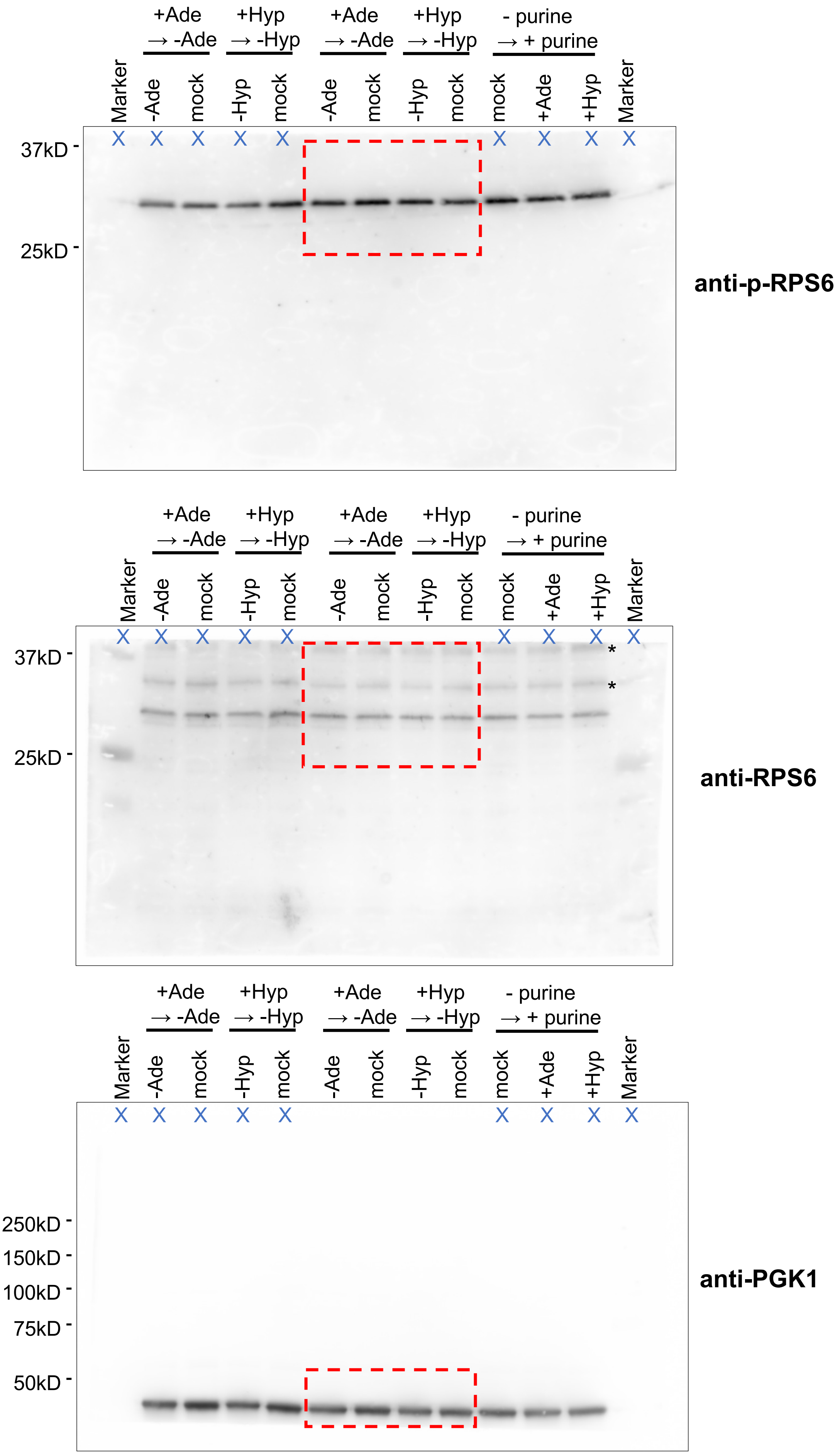

*, a non-specific band in cell extract.

S13C Fig

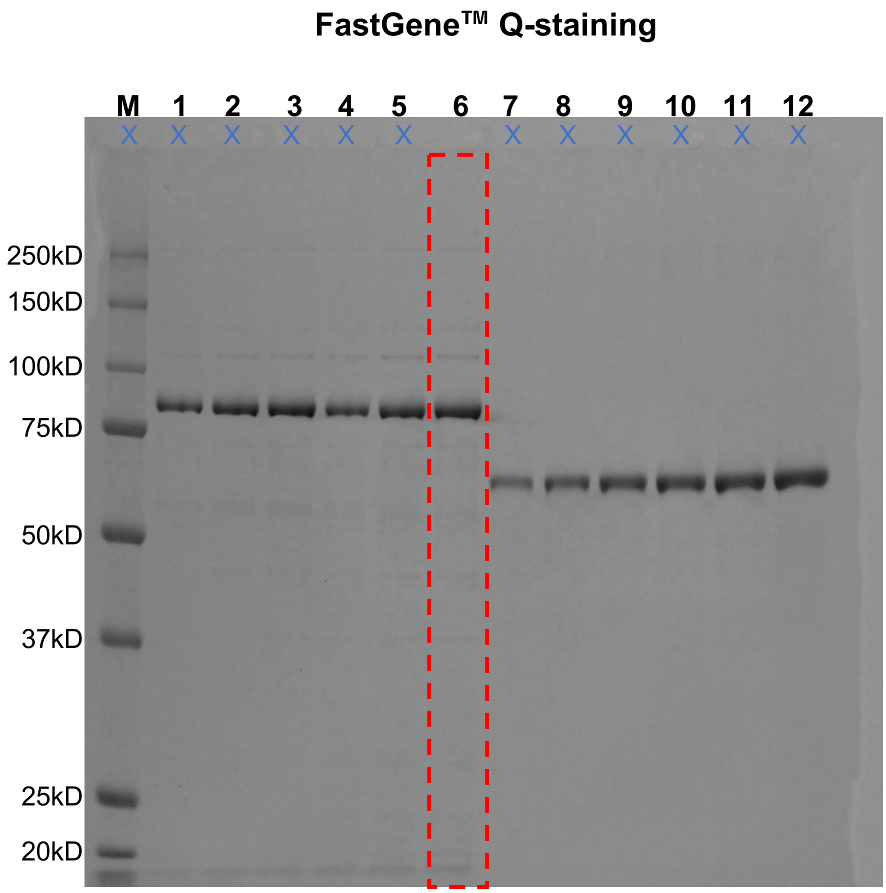

M: Precision plus protein dual color standards (Bio-Rad)

1-3: Purified Ade4-mNG: 0.5, 1.0, 1.5 µl

4-6: Purified Ade4-D373A/D374A-mNG: 0.5, 1.0, 1.5 µl

7-12: BSA: 0.2, 0.3, 0.4, 0.5, 0.6, 0.7 µg

S15E Fig

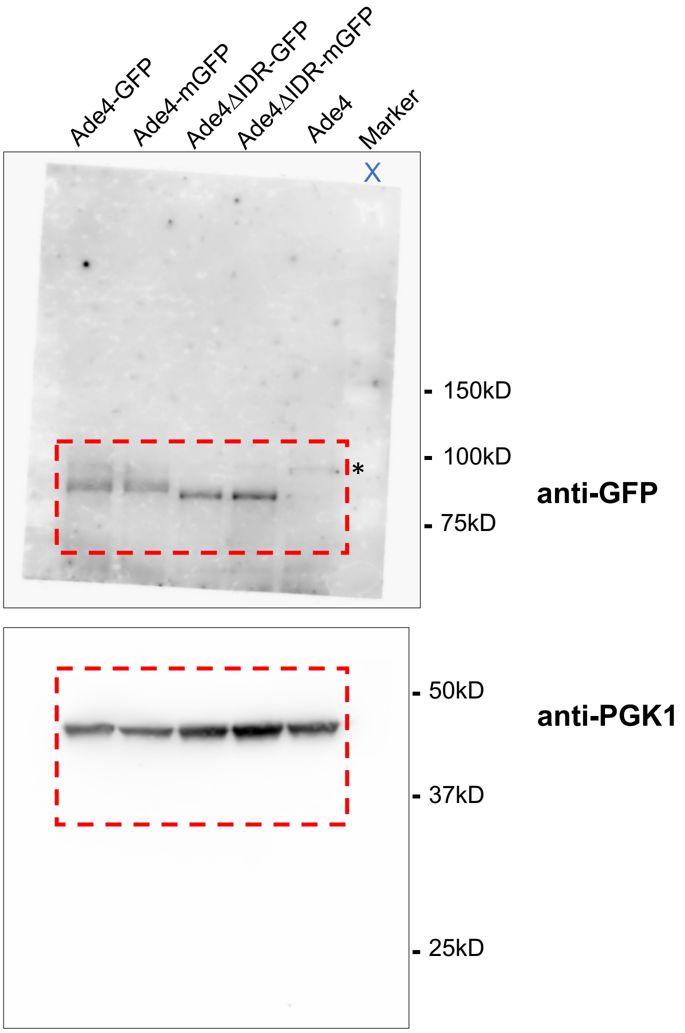

*, a non-specific band.
