# Supplementary material for: Phase separation of the PRPP amidotransferase into dynamic condensates promotes de novo purine synthesis in yeast
Source: PLoS Biol. 2025 Apr 10;23(4):e3003111. doi: 10.1371/journal.pbio.3003111 (PMC12017579; doi:10.1371/journal.pbio.3003111)
Supplement: Attachment — Submitted filename: Response_v5.pdf [file pbio.3003111.s035.pdf]

Manuscript ID: PBIOLOGY-D-24-02276R1
Response to the Reviewers

Dear Dr. Alvarez-Garcia and the Academic Editor:

We sincerely thank the Reviewer and the Academic Editor for their valuable and constructive comments. We performed additional experiments and revised the manuscript according to their suggestions. Point-by-point responses to each comment below are highlighted in blue, while the Reviewer's and Editor's comments are in italics. Major revised contents in the revised manuscript are also indicated in blue font.

Reviewer's comments

Reviewer 1:

In this interesting study, Takaine et al investigate how the S. cerevisiae PRPP amidotransferase Ade4 is regulated in different nutrient conditions. They describe that Ade4 forms highly dynamic and reversible foci upon purine starvation. Formation of these foci can be prevented by deletion of a conserved IDR region in the protein or by addition of hexane diol, leading the authors to conclude that the observed foci represent liquid condensates. These condensates seem to depend on high levels of intracellular crowding as Ade4 focus formation depends on TORC1 induced ribosome biogenesis and synthetic crowding agents (PEG, Ficoll) induce purified Ade4 to undergo a phase transition in vitro. Furthermore, the authors describe how metabolic changes that occur in response to purine starvation promote Ade4 condensation. In vitro, Ade4 condensation is strongly increased by binding to its substrate PRPP and slightly inhibited by AMP. At high crowding levels the positive effect of PRPP is however only modest. To assess the functional relevance of the identified metabolite- and crowding dependent condensation of Ade4, the authors performed molecular dynamics simulations that suggest that condensation supports substrate channeling between the active sites of Ade4. I am not qualified to assess the quality of these molecular dynamics calculations. Finally, the authors show that deletion of the IDR domain in Ade4 reduces cell fitness in purine depleted conditions.

Overall, this is an exciting manuscript with an impressive amount data. The experimental data are of high quality and generally support the author's conclusions. I have a number of points the authors might want to address before publication

We sincerely thank Reviewer 1 for finding our work interesting and for the insightful suggestions. We followed these suggestions and consider the results obtained from the additional experiments and the associated discussions to have significantly improved the manuscript.

1. From the observation that Ade4 focus formation depends on TORC1 and Sfp1 activity, the authors conclude that ribosome biogenesis dependent crowding is needed for condensate formation. A recent study by the Holt lab (PMID: 39059370) suggests that polysomes rather than individual ribosomes play a major role in cytoplasmic crowding. As polysomes collapse much faster than overall ribosome concentration decreases, it would be interesting to see how quickly Ade4 condensates disassemble upon Rapamycin treatment and whether it can be prevented by simultaneous treatment with Cycloheximide (prevents polysome collapse).

Response: We appreciate the Reviewer's suggestion and investigated the disassembly of Ade4 condensates upon the treatment with rapamycin. Using Ade4-GFP foci as markers, we observed a rapid decrease in the percentage of cells with foci immediately after the rapamycin treatment, with nearly complete disassembly within 30 min (Fig R1A and R1B). The effects of the mock treatment with DMSO were negligible. These results are consistent with the findings reported by Xie et al. (2024) (PMID: 39059370) [1], which suggest that polysomes, collapsing within 15 min of the rapamycin treatment, plays a major role in molecular crowding in yeast cells, whereas autophagy-dependent ribosome degradation requires more than 1 hour.

Unexpectedly, the treatment with cycloheximide alone also caused the disassembly of Ade4-GFP foci ("CHX" in Fig R1C), albeit more gradually than rapamycin. Cycloheximide may indirectly dissolve Ade4 condensates by changing intracellular PRPP or purine nucleotide levels. The simultaneous treatment with cycloheximide and rapamycin also reduced the percentage of foci over time ("CHX+Rapa" in Fig R1C). We were unable to conclusively establish whether cycloheximide counteracted rapamycin by preventing polysome collapse because the results of the simultaneous treatment varied with the treatment time (in the same experiment) and between experiments (note that Fig R1 shows only an example). Cycloheximide may inhibit polysome collapse; however, this effect may be masked by its synergistic interaction with rapamycin.

We also examined the effects of diazaborine on Ade4 condensates. The treatment with diazaborine resulted in the disassembly of Ade4-GFP foci, although at a slower

rate than with rapamycin (Fig R1A and R1B). These results suggest that TORC1 activity and ribosome synthesis are required not only for the assembly, but also the maintenance of Ade4 condensates. The results from these experiments, except for the cycloheximide/rapamycin co-treatment, are included in S4 Fig, and corresponding text has been added to the Results section of the revised manuscript (lines 205-211).

Since mechanistic analyses of intracellular molecular crowding effects were beyond the scope of this study, and rescue experiments with cycloheximide yielded inconclusive results, we refrained from concluding that polysomes are solely responsible for the molecular crowding effects that drive Ade4 condensate formation. Instead, we mentioned in the Discussion section (lines 586-597) that polysomes may contribute to cytoplasmic crowding, as suggested by Xie et al. (2024). Previous studies showed that a treatment with diazaborine significantly reduced both 60S ribosomal subunits and polysomes [2] [3], demonstrating that polysome abundance depends on ribosome levels and the difficulty of analyzing these two factors independently.

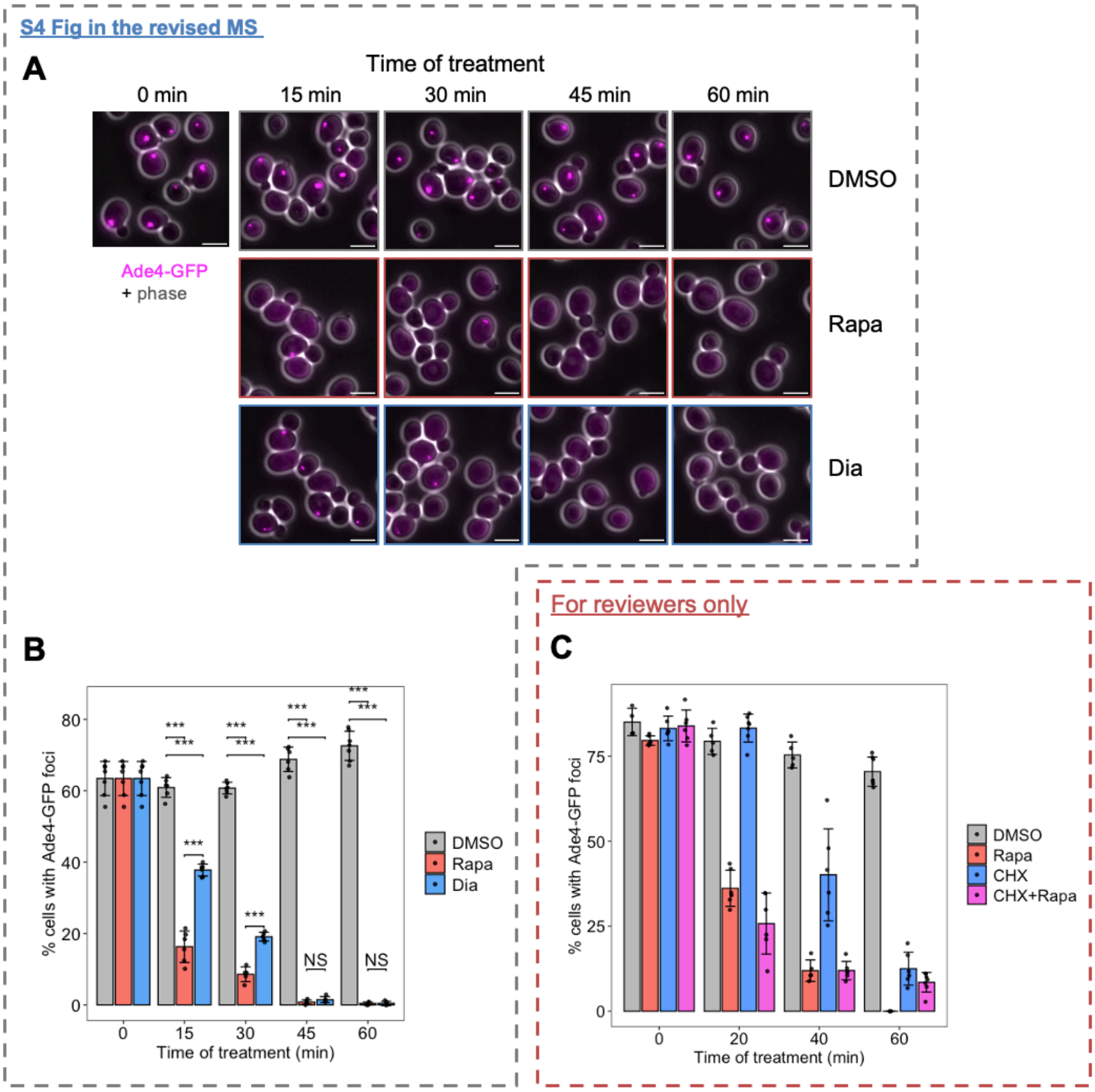

Reviewer Figure R1: TORC1 activity and ribosome synthesis are required for the maintenance of Ade4 condensation

A. Cells with Ade4-GFP foci in the absence of purine bases were supplemented with 0.02% (v/v) DMSO (DMSO), 0.02% DMSO plus 1 μg/ml rapamycin (Rapa), or 0.02% DMSO plus 5 μg/ml diazaborine (Dia) and then imaged every 15 min for 1 h. Representative cell images are shown. Scale bar = 5 μm.

B. Quantification of data shown in (A). The percentage of cells with foci per one field of view were quantified and plotted. Bars and error bars indicate the mean ± 1SD. More than 5 fields of view were imaged for each condition and approximately 200 cells were examined per field. Note that data at time = 0 are identical across the three groups because untreated cells were imaged and then divided into three subgroups that were supplemented with each drug. *P*-values were calculated using the two-tailed Tukey-Kramer's multiple comparison test. ***, $p < 10^{-4}$.

C. Cycloheximide dissolves Ade4-GFP foci. Cells with Ade4-GFP foci in the absence of purine bases were supplemented with 0.02% (v/v) DMSO (DMSO), 0.02% DMSO plus 1 μg/ml rapamycin (Rapa), 0.02% DMSO plus 100 μg/ml cycloheximide (CHX), or 0.02% DMSO plus 100 μg/ml cycloheximide and 1 μg/ml rapamycin (CHX+Rapa) and then imaged every 20 min for 1 h. The percentages of cells with foci per one field of view were quantified and plotted. Bars and error bars indicate the mean ± 1SD. Four to seven fields of view were imaged under each condition, with approximately 100 cells examined per field of view. An example of the results obtained is shown.

---

2. While PRPP can boost condensate formation at intermediate PEG concentrations, at high concentrations (10%) PRPP is much less potent at inducing Ade4 condensation. Whether or not PRPP levels are important in regulating Ade4 condensates therefore depends on the level of crowding in the cytoplasm. It would be useful if the authors could relate the PEG concentration to levels of intracellular crowding.

Response: We appreciate the Reviewer's insightful comment. We propose that the effect of PRPP in inducing Ade4 condensation is not diminished, but rather obscured at 10% PEG8000 (hereafter, PEG8K). At this concentration, the molecular crowding effect is strong enough to cause Ade4-mNG to condense without PRPP, even in the presence of AMP, making the effect of PRPP difficult to quantify. In contrast, at 7.5% PEG8K, Ade4-mNG requires PRPP to condense, allowing the effect to be more clearly observed. In Fig 7A-7C, we aimed to demonstrate that PRPP enhances Ade4 condensation in 10% PEG8K (where spontaneous condensation occurs) and also promotes Ade4 condensate formation in 7.5% PEG8K (where spontaneous condensation does not occur).

However, we acknowledge that the text in the original manuscript may have been unclear. We clarified this in the revised manuscript (lines 360-365) as follows: "These results suggest that PRPP not only enhances Ade4 condensation under condensing conditions (≥10% PEG8K), but also promotes the formation of Ade4 condensates under non-condensing conditions (≤7.5% PEG8K). Furthermore, the inhibitory effect of 1 mM AMP on Ade4 condensation was effectively counteracted by PRPP at concentrations higher than 50 μM in both 7.5% PEG8K and 10% PEG8K (Figs 7D, 7E, and S12D), suggesting that PRPP antagonizes the inhibitory effect of AMP."

In response to the Reviewer's suggestion to relate PEG concentrations to cytoplasmic crowding, we conducted additional experiments with 5% PEG8K (Fig R2).

S12E-S12G Fig in the revised MS

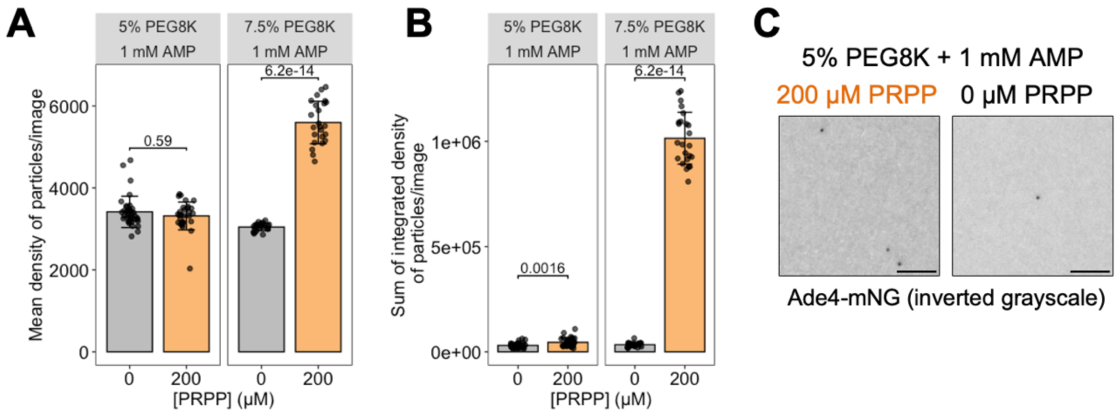

Reviewer Figure R2: PRPP fails to counteract the inhibitory effect of AMP in 5% PEG8K

In the presence of 1 mM AMP and the indicated concentrations of PRPP, 0.2 μM Ade4-mNG was supplemented with 5% or 7.5% PEG8K. From 2-3 independent preparations, 24-36 fields of view were imaged for each condition. (A) The mean fluorescence intensities of Ade4-mNG condensates were averaged per field and plotted. Throughout the figure, bars and error bars indicate the mean ± 1SD. (B) The integrated fluorescence intensities of the condensates were summed per field and plotted. *P*-values were calculated using the two-tailed Mann-Whitney U test. Representative images under 5% PEG8K conditions are shown in (C). Scale bars = 5 μm. Note that although PRPP increased the number of extremely small condensates in 5% PEG8K, it failed to strongly promote the condensation of Ade4-mNG.

In 5% PEG8K and 1 mM AMP, the addition of 200 μM PRPP caused a small, but significant increase in the integrated fluorescence intensity of condensates (Fig R2A and R2B), but did not significantly promote further condensation (Fig R2C). These results differ from those observed with 7.5% PEG8K (Fig 7D and 7E and Fig R2A and R2B) and suggest that the molecular crowding effect of 5% PEG8K is too weak for PRPP to counteract the inhibitory effect of AMP.

We included these results in the revised manuscript as S12E-S12G Fig and added a corresponding sentence to the Results section (lines 366-367). Additionally, we summarized the conditions for Ade4 condensation in the table as Fig R3 (S16 Fig in the revised manuscript).

S16 Fig in the revised MS

Summary of conditions for Ade4 condensation

	In vitro				*In vivo*	
	PEG8K				TORC1	
	2.5%	5%	7.5%	10%	inactive	active
- PRPP	- (ND)	- (-)	+/- (-)	+ (+)	-	-
+ PRPP	- (ND)	+ (-)	+ (+)	+ (+)	-	+

"-", condensates are barely detectable; "+", considerable amounts of condensates are detectable; "+/-", small amount of condensates are detectable; ND, not determined.
Marks in parenthese are results in the presence of 1 mM AMP.

Reviewer Figure R3: Summary of conditions for Ade4 condensation

In vivo results are based on data shown in Figs 1, 2, and 4. *In vitro* results are based on data shown in Figs. 3C, 6C, 7A, 7B, and 7D and the new result (Reviewer Fig R2).

The table indicates that the 7.5% PEG8K condition most accurately reflects the regulation of Ade4 condensation in cells. At 10% PEG8K, the molecular crowding effect is so strong that condensation occurs independently of PRPP or AMP. In contrast, at 2.5% PEG8K, the crowding effect is too weak for PRPP to induce Ade4 condensation, resembling the intracellular crowding conditions in cells treated with rapamycin or diazaborine. At 5% PEG8K, PRPP regulates the condensation of Ade4 in the absence of AMP; however, since millimolar levels of purine nucleotides are present in cells, regulation that ignores their inhibitory effect is physiologically irrelevant. We therefore conclude that the molecular crowding effect in the cytoplasm of actively growing budding yeast cells with active TORC1-dependent ribosome synthesis corresponds to approximately 7.5% PEG8K. Appropriate crowding conditions, neither too strong nor too weak, are necessary for the regulation of Ade4 condensation by PRPP. A corresponding paragraph has been added to the Discussion section of the revised manuscript (lines 606-619).

3. It would help if the negative control for the in vitro condensation experiments (mNG, Figure S4B) would be shown next to the Ade4-mNG condensates in the main figure

and displayed in the same way (inverted intensities).

Response: We appreciate the suggestion and presented original Fig S4B as Fig 3D in the revised manuscript and revised the text accordingly (lines 232-233). We also replaced data with those that more clearly show that mNG alone did not form *in vitro* particles even in 10% PEG8K.

4. The authors show that ADE4-IDRΔ mutants display a growth defect on ADE- which is accentuated in the absence of ammonium. This is consistent with the idea that Ade4 condensation promotes purine synthesis by substrate channeling. Another possible interpretation of these data is that deleting the IDR domain reduces the intrinsic enzymatic activity of Ade4 and that the observed growth defect on Ade- is caused by this mutation. Do the authors have data to exclude this possibility. If not, they should at least discuss this possibility.

Response: We appreciate and totally agree with this comment, acknowledging it as a limitation of the present study. While we hypothesize that the IDR, being separate from the enzymatic domains, does not directly impair enzymatic activity, we cannot confirm this without direct enzymatic activity measurements. Unfortunately, our attempts to compare the enzymatic activities of Ade4 and Ade4ΔIDR were unsuccessful. Therefore, we cannot distinguish whether the slower growth of the Ade4ΔIDR mutant is due to reduced enzymatic activity, the absence of activation through condensation-dependent mechanisms, or both.

To address this, we examined the relationship between the aggregation state of Ade4ΔIDR and *de novo* purine synthesis. Specifically, we compared the growth of cells expressing Ade4ΔIDR-GFP and Ade4ΔIDR-mGFP (Fig R4A-R4C). Since GFP and mGFP differ in their dimerization constants [4], GFP dimerization may artificially promote Ade4 aggregation, as we demonstrated in Fig 1B and 1C. Growth rates in the absence of adenine were in the following order: Ade4-GFP ≳ Ade4-mGFP > Ade4ΔIDR-GFP > Ade4ΔIDR-mGFP (Fig R4A-R4C). While the difference between Ade4-GFP and Ade4-mGFP was not significant, the difference between Ade4ΔIDR-GFP and Ade4ΔIDR-mGFP was significant (Fig R4C and R4D), despite similar expression levels between constructs (Fig R4E). These results suggest that the

aggregation-promoting property of GFP partially rescues the growth delay phenotype of the *ADE4ΔIDR* mutant. We included the new results in S15 Fig and added a corresponding paragraph to the Results section in the revised manuscript (lines 462-474).

Since Ade4ΔIDR-GFP and Ade4ΔIDR-mGFP (as well as Ade4-GFP and Ade4-mGFP) differ by only a single amino acid in the GFP moiety, their intrinsic enzymatic activities may theoretically be identical. Given that Ade4ΔIDR-GFP likely forms multimers more readily than Ade4ΔIDR-mGFP, the present results strongly support the hypothesis that Ade4 condensation, even when artificially induced, promotes *de novo* purine synthesis. The lack of a significant difference between mGFP and GFP in full-length Ade4 may be due to wild-type Ade4 condensation and enzymatic activation already being optimized. We added a paragraph discussing these results to the end of the Discussion section in the revised manuscript (lines 630-640).

S15 Fig in the revised MS

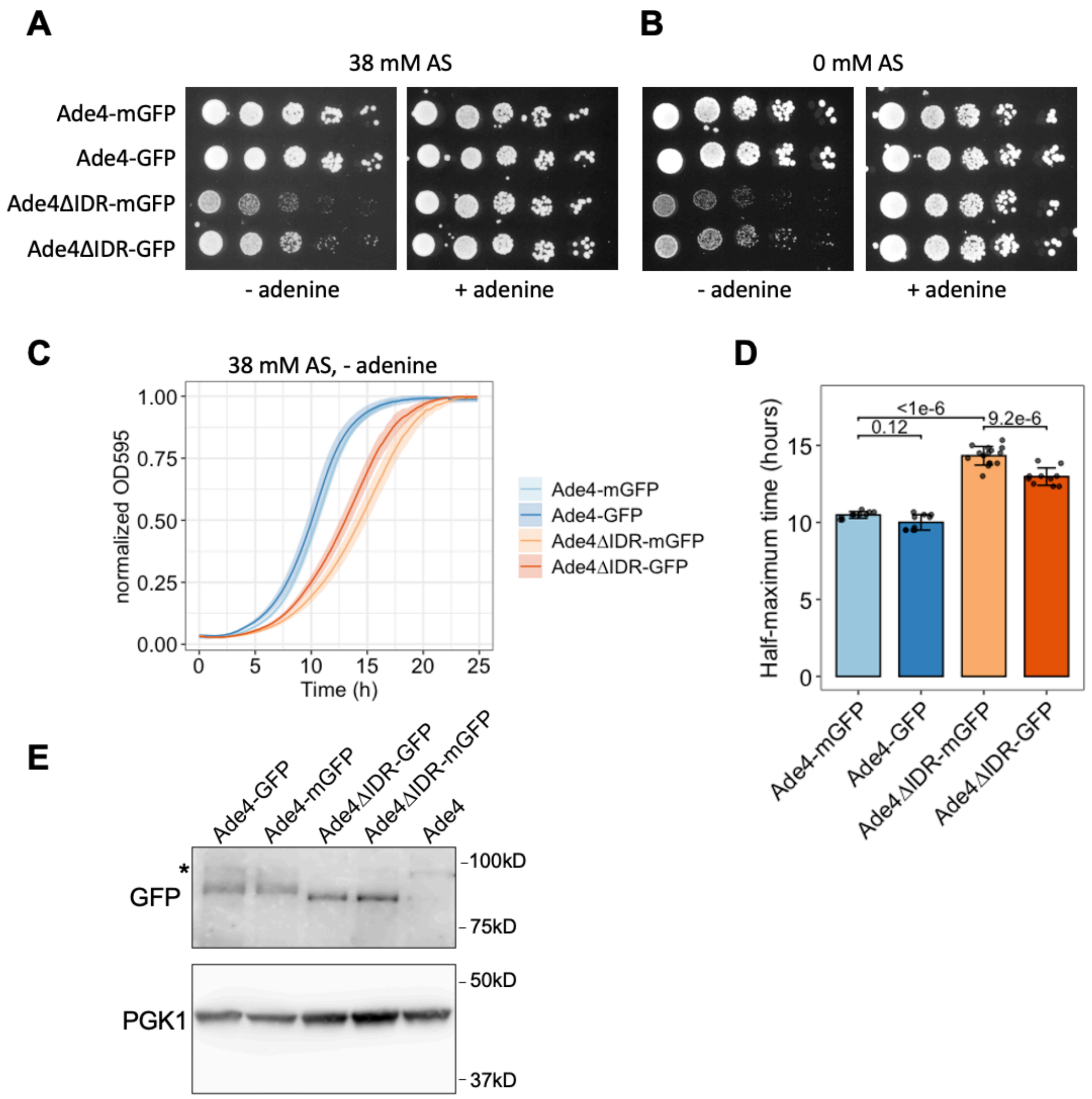

Reviewer Figure R4: **The formation of Ade4 condensates is important for efficient *de novo* purine synthesis during cell growth, related to Fig 9**

A-B. Growth of cells expressing Ade4-mGFP, Ade4-GFP, Ade4ΔIDR-mGFP, and Ade4ΔIDR-GFP on solid media. Cells of each strain was serially diluted (five-fold), spotted on 2% glucose medium containing 38 mM ammonium sulfate (A) and 8 mM proline (no ammonium sulfate) (B), and then grown at 30°C for 2 days.

C. Growth curves of cells expressing Ade4-mGFP, Ade4-GFP, Ade4ΔIDR-mGFP, and Ade4ΔIDR-GFP in liquid media without adenine. Cells were grown at 30°C for 25 h in medium containing the indicated nitrogen source in the absence of adenine. Data are the mean of 10-15 replicates, and the shaded area indicates ± 1SD.

D. Quantification of data shown in (C). The time to half-maximum density (corresponding to

normalized OD_{595} = 0.5) was calculated and plotted. Bars and error bars indicate the mean ± 1SD. *P*-values were calculated using the Tukey-Kramer multiple comparison test.

E. Immunoblot analysis of the protein level of Ade4 tagged C-terminally with GFP or mGFP. Total cell extracts were prepared from cells expressing the indicated Ade4 construct. Ade4 proteins were detected as bands at approximately 80 kDa by an anti-GFP antibody. The asterisk indicates a non-specific band. The bands of PGK1 were shown as a loading control.

Comments from the Academic Editor

I share reviewer 1's enthusiasm for this manuscript and would like to see the authors address their comments. I additionally have a couple of requests:

We really appreciate the positive response and important suggestions from the Academic Editor. We followed these suggestions and consider the new results obtained, particularly from additional experiments on monitoring TORC1 activity, to have strengthened support for the hypothesis of the manuscript.

i/ Line 245: …However, environmental purine bases, including adenine, do not affect TORC1 activity in mammalian cells 29, 30. I don't think these references come to this conclusion. I would recommend determining if TORC1 activity is affected by the treatments performed in figure 5B. This may be particularly important given that phosphorylation of Prs5 is potentially regulated downstream of TORC1 (DOI: 10.1101/gad.532109).

Response: We appreciate the suggestion and examined whether TORC1 activity is affected by the presence of purine bases in the medium. To monitor TORC1 activity, we analyzed the phosphorylation of 40S ribosomal protein S6 (Rps6) at Ser235/Ser236 using an immunoblot analysis because these TORC1-mediated phosphorylation sites are highly conserved and are widely used to assess TORC1 activity across species [5]. We successfully detected total Rps6 and phosphorylated Rps6 (p-Rps6); however, some non-specific bands were observed (asterisks in Fig R5A-R5C). The treatment with rapamycin significantly reduced the intensity of the p-Rps6 band and caused a slight downward shift in the total Rps6 band, confirming that phosphorylation at this site is regulated by TORC1 activity (Fig R5A). This downward shift in total Rps6 upon TORC1 inactivation was previously reported (*e.g.*, Figs 1B and 3A in Yerlikaya et al. (2015) [6]).

In contrast to rapamycin, supplementation with adenine or hypoxanthine did not significantly affect the phosphorylation state of Rps6 (Fig R5B). We also examined the effect of purine base removal and found that the phosphorylation state of Rps6 remained largely unchanged (Fig R5C). These results suggest that the addition or removal of purine bases does not affect TORC1 activity in yeast cells, at least for short periods (30-40 min). We included these results as S7 Fig in the revised manuscript, added a corresponding paragraph to the Results section (lines 252-269), and described the protocol for the immunoblotting analysis using anti-Rps6 and anti-p-Rps6

antibodies in the Materials and Methods section (lines 742-751).

S7 Fig in the revised MS

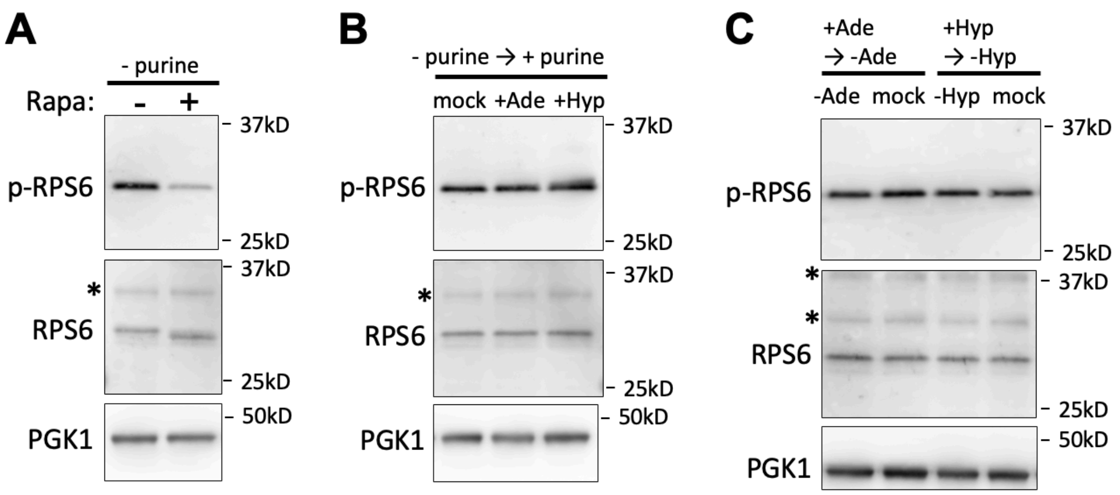

Reviewer Figure R5: **Effect of environmental purine bases on TORC1 activity in yeast cells**

A. Immunoblot analysis of the phosphorylation state of Rps6. Wild-type (BY4741) cells grown in the absence of purine bases were supplemented with 0.005% (v/v) DMSO (Rapa: -) or 0.005% DMSO plus 0.5 µg/ml rapamycin (Rapa: +) for 30 min, and total cell extracts were prepared. Total Rps6 (Rps6) and phosphorylated Rps6 (p-Rps6) were detected as bands at approximately 30 kDa. The bands of Pgk1 were shown as a loading control. Throughout the figure, asterisks indicate non-specific bands.

B. Supplementation with purine bases did not markedly affect the phosphorylation state of Rps6. Wild-type cells grown in the absence of purine bases were supplemented with 20 µg/ml adenine (+Ade) or 25 µg/ml hypoxanthine (+Hyp) for 30 min, and total cell extracts were prepared. The phosphorylation of Rps6 was analyzed by immunoblotting as described in (A).

C. The removal of purine bases did not markedly affect the phosphorylation state of Rps6. Wild-type cells grown in the presence of 20 µg/ml adenine (+Ade) or 25 µg/ml hypoxanthine (+Hyp) were washed three times with the same medium (mock) or the medium lacking purine bases (-Ade, -Hyp), cultured for 40 min, and total cell extracts were prepared. The phosphorylation of Rps6 was analyzed by immunoblotting as described in (A).

Regarding the cited references, Hoxhaj et al [7] and Emmanuel et al. [8] (refs. 29 and 30 in the original manuscript) included observations of TORC1 activity in the presence of adenine, hypoxanthine, or guanine. Hoxhaj et al. evaluated TORC1 activity by S6K phosphorylation, while Emmanuel et al. used the phosphorylation of S6K or

Rps6. Although the effect of purine bases on TORC1 activity was not a primary focus in these studies, the findings obtained demonstrate that the addition of purine bases (adenine, guanine, or inosine) did not markedly affect TORC1 activity in mammalian cells (e.g., Figs 7G, 7H, S2A, S2D, and S5H-I in Hoxhaj et al.; Figs 1D, 1F, 2B, 2F, 2H, 4C, 5B, S4A, S5B, and S6B in Emmanuel et al.). We revised the sentence pointed out by the Academic Editor to convey the above situation more clearly in the revised manuscript (lines 252-254) as follows: "Previous findings [7, 8] suggest that environmental purine bases do not affect TORC1 activity in mammalian cells (although this was not the main focus of these studies).".

ii/ The molecular dynamics data are quite compelling, but I would like to see these hypotheses confirmed in vivo if possible. For example, does the Arg333Ala mutant have any phenotypes in yeast? Could it perhaps rescue the slow growth phenotype when introduced into the Ade4ΔIDR-3HA gene?

Response: We appreciate the suggestion. Since the Arg residue is conserved in Ade4 (Fig R6A), we introduced the Arg333Ala mutation (Arg339Ala in budding yeast) into the *ADE4-3HA* and *ADE4-mNG* strains. Unfortunately, the *ade4-R339A* mutants showed adenine auxotrophy, indicating that the mutation compromised Ade4 activity *in vivo* (Fig R6B). As expected, combining the R339A mutation with ΔIDR did not restore the slow growth phenotype of the *ADE4ΔIDR* mutant in the absence of adenine (Fig R6B). Moreover, Ade4-R339A-mNG failed to form detectable punctate structures (Fig R6C). A previous study reported that Arg333 was critical for the enzymatic activity of *E. coli* PPAT [9] because it interacts with PRPP through hydrogen bonds (also shown in Fig 8G of our manuscript). This result supports our result showing that the R339A mutation likely disrupted PRPP binding, inactivating the enzyme and impairing PRPP-dependent condensate formation.

Regarding further confirmation of the hypothesis that Ade4 condensation enhances *de novo* purine synthesis, refer to our response to Reviewer 1, comment #4. We provided novel results demonstrating that the slow growth phenotype of the *ADE4ΔIDR* mutant may be partially restored by artificially inducing the aggregation of the mutant protein.

For reviewers only

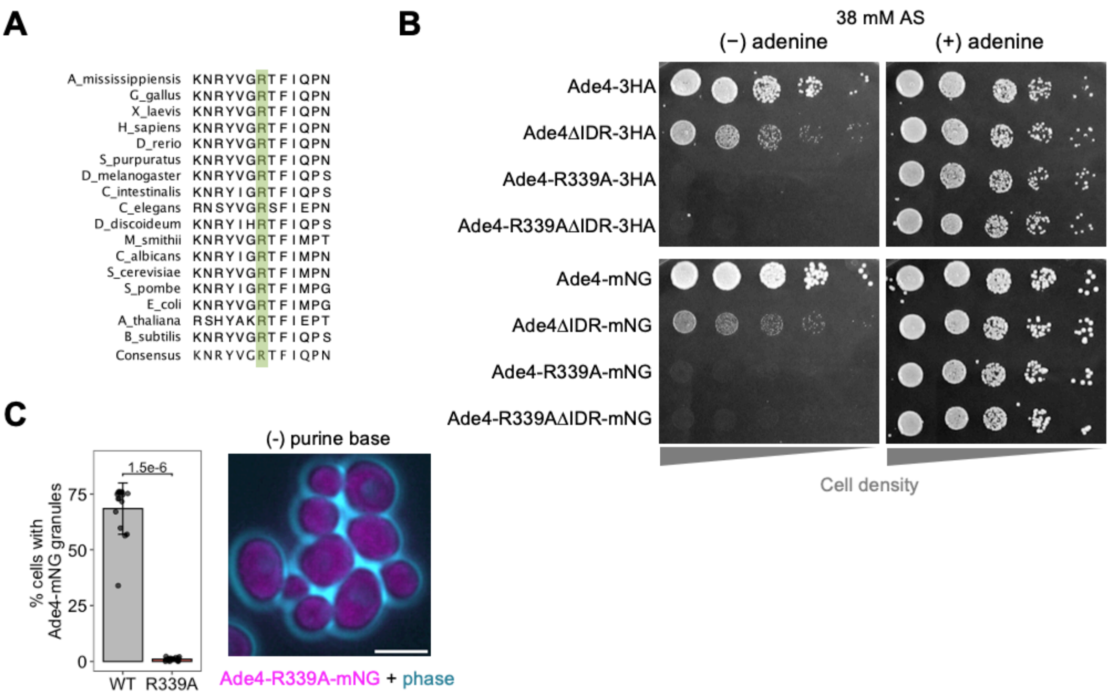

Reviewer Figure R6: **Characterization of *ade4-R339A* mutants**

A. The R333 residue of *E. coli* PPAT is well conserved among PPATs from different species.

B. Growth of cells expressing Ade4-R339A on solid media. Cells expressing the indicated constructs were serially diluted (five-fold), spotted on 2% glucose medium containing 38 mM ammonium sulfate, and then grown at 30°C for 1.5-2 days.

C. R339 is important for condensate assembly. Cells expressing Ade4-mNG (WT) and Ade4-R339A-mNG (R339A) were incubated in the absence of purine bases for 45 min and imaged. The percentages of cells with granules per one field of view were quantified and plotted. Bars and error bars indicate the mean ± 1SD. Data were pooled from 2 independent experiments, and 16 fields of view were imaged for each condition. *P*-values were calculated using the two-tailed Mann-Whitney U test. A representative image of the mutant cells is shown. Scale bar = 5 µm.

References:

1. Xie Y, Shu T, Liu T, Spindler MC, Mahamid J, Hocky GM, et al. Polysome collapse and RNA condensation fluidize the cytoplasm. Mol Cell. 2024;84(14):2698-716 e9. doi: 10.1016/j.molcel.2024.06.024. PubMed PMID: 39059370; PubMed Central PMCID: PMCPMC11539954.

2. Pertschy B, Zisser G, Schein H, Köffel R, Rauch G, Grillitsch K, et al. Diazaborine Treatment of Yeast Cells Inhibits Maturation of the 60S Ribosomal Subunit. Molecular and Cellular Biology. 2004;24(14):6476-87. doi: 10.1128/MCB.24.14.6476-6487.2004.
3. Steffen KK, MacKay VL, Kerr EO, Tsuchiya M, Hu D, Fox LA, et al. Yeast Life Span Extension by Depletion of 60S Ribosomal Subunits Is Mediated by Gcn4. Cell. 2008;133(2):292-302. doi: 10.1016/j.cell.2008.02.037.
4. Zacharias DA, Violin JD, Newton AC, Tsien RY. Partitioning of lipid-modified monomeric GFPs into membrane microdomains of live cells. Science. 2002;296(5569):913-6. doi: 10.1126/science.1068539. PubMed PMID: 11988576.
5. González A, Shimobayashi M, Eisenberg T, Merle DA, Pendl T, Hall MN, et al. TORC1 Promotes Phosphorylation of Ribosomal Protein S6 via the AGC Kinase Ypk3 in Saccharomyces cerevisiae. PLOS ONE. 2015;10(3):e0120250. doi: 10.1371/journal.pone.0120250.
6. Yerlikaya S, Meusburger M, Kumari R, Huber A, Anrather D, Costanzo M, et al. TORC1 and TORC2 work together to regulate ribosomal protein S6 phosphorylation in Saccharomyces cerevisiae. Molecular Biology of the Cell. 2015;27(2):397-409. doi: 10.1091/mbc.e15-08-0594.
7. Hoxhaj G, Hughes-Hallett J, Timson RC, Ilagan E, Yuan M, Asara JM, et al. The mTORC1 Signaling Network Senses Changes in Cellular Purine Nucleotide Levels. Cell Rep. 2017;21(5):1331-46. doi: 10.1016/j.celrep.2017.10.029. PubMed PMID: 29091770; PubMed Central PMCID: PMCPMC5689476.
8. Emmanuel N, Ragunathan S, Shan Q, Wang F, Giannakou A, Huser N, et al. Purine Nucleotide Availability Regulates mTORC1 Activity through the Rheb GTPase. Cell Rep. 2017;19(13):2665-80. doi: 10.1016/j.celrep.2017.05.043. PubMed PMID: 28658616.
9. Bera AK, Smith JL, Zalkin H. Dual role for the glutamine phosphoribosylpyrophosphate amidotransferase ammonia channel. Interdomain signaling and intermediate channeling. J Biol Chem. 2000;275(11):7975-9. Epub 2000/03/14. doi: 10.1074/jbc.275.11.7975. PubMed PMID: 10713115.
